# Genetically-Encoded Fluorescence Barcodes for Single-Cell Analysis

**DOI:** 10.1101/2024.10.23.619855

**Published:** 2024-10-24

**Authors:** Xiaoming Lu, Daniel J. Pritko, Megan E. Abravanel, Jonah R. Huggins, Feranmi Ogunleye, Tirthankar Biswas, Katia C. Ashy, Semaj K. Woods, Mariclaire W.T. Livingston, Mark A. Blenner, Marc R. Birtwistle

**Affiliations:** 1 Department of Chemical and Biomolecular Engineering, Clemson University; 2 Department of Chemical and Biomolecular Engineering, University of Delaware

## Abstract

Genetically-encoded, single-cell barcodes are broadly useful for experimental tasks such as lineage tracing or genetic screens. For such applications, a barcode library would ideally have high diversity (many unique barcodes), non-destructive identification (repeated measurements in the same cells or population), and fast, inexpensive readout (many cells and conditions). Current nucleic acid barcoding methods generate high diversity but require destructive and slow/expensive readout, and current fluorescence barcoding methods are non-destructive, fast, and inexpensive to readout but lack high diversity. We recently proposed theory for how fluorescent protein combinations may generate a high-diversity barcode library with non-destructive, fast and inexpensive identification. Here, we present an initial experimental proof-of-concept by generating a library of ~150 barcodes from two-way combinations of 18 fluorescent proteins. We use a pooled cloning strategy to generate a barcode library that is validated to contain every possible combination of the 18 fluorescent proteins. Experimental results using single mammalian cells and spectral flow cytometry demonstrate excellent classification performance of individual fluorescent proteins, with the exception of mTFP1, and of most evaluated barcodes, with many true positive rates >99%. The library is compatible with genetic screening for hundreds of genes (or gene pairs) and lineage tracing hundreds of clones. This work lays a foundation for greater diversity libraries (potentially ~10^5^ and more) generated from hundreds of spectrally-resolvable tandem fluorescent protein probes.

## Introduction

Cell barcoding, a collection of methods by which single cells (or sometimes populations) are uniquely labeled, has been and remains fundamental to answering a wide variety of biological questions^1–15^. Applications include studying cell type and/or state dynamics (e.g. evolution of tumor heterogeneity^15–18^), tracking lineage (e.g. cell fate decisions^4,14,15,19,20^), or identifying genetic perturbations in a cell (e.g. CRISPR screening)^12,21–26^. Barcodes are usually made from either fluorophores (small molecules or fluorescent proteins) or nucleic acids, with each having benefits and drawbacks depending on the questions at hand^1–4,6,7,22,25–34^. In general, an ideal barcode library has high diversity (many unique barcodes), is fast/inexpensive to readout (analyze many cells and conditions), and its observation is non-destructive (repeated measurements of the same cell or population).

Fluorescent barcodes can be generated from combinations of fluorophores (fluorescent proteins or small molecules), with additional diversity sometimes possible from an intensity dimension^1,3,4,20,27,35–37^. Fluorescent barcodes are usually non-destructive/fast/inexpensive to readout via flow cytometry or microscopy. However, they to-date lack high diversity relative to nucleic acid approaches or genome scale. Small molecule fluorophore approaches have not yet been used to identify genetic perturbations^1,3,27^. Fluorescent protein (fp) barcodes are genetically-encoded but have even less diversity^4,20,36,37^. Alternatively, nucleic acid barcodes are usually random DNA sequences^25,26,28–34^, which gives incredible diversity. For example, just 10 bases can generate ~10^6^ (4^10^) barcodes. However, readout is often single-cell (or bulk) sequencing, which is destructive and often much slower and more expensive than flow cytometry or microscopy. These properties prevent observation of the cell after barcode identification and hinder large-scale experiments, although have the benefit of often providing the transcriptome^1,21^.

We recently proposed theory for and simulations studies supporting a fluorescent protein-based, single-cell barcoding method that bridges fast, non-destructive fluorescence readouts with the large barcode diversity^38^. The barcoding approach is based on Multiplexing using Spectral Imaging and Combinatorics (MuSIC)^27,38–40^, which generates unique emission spectra signatures from combinations of individual fluorescent proteins. In this paper, we demonstrate experimental proof-of-principle by generating ~150 barcodes from 18 fluorescent proteins. We use a pooled cloning strategy to generate a barcode library that is validated to contain every possible combination of the 18 fluorescent proteins. Experimental results using single mammalian cells and spectral flow cytometry demonstrate excellent classification performance of individual fluorescent proteins, with the exception of mTFP1, and of most evaluated barcodes, with many true positive rates >99%. The library is compatible with genetic screening for hundreds of genes (or gene pairs) and lineage tracing hundreds of clones. This work lays a foundation for greater diversity libraries (potentially 10^5^ and more) generated from hundreds of spectrally-resolvable tandem fluorescent protein probes.

## Results

### MuSIC Barcodes in Single Cells

While previous work explored the concept of a MuSIC barcode and their application to high-dimensional single cell analysis^38^, experimental demonstration remained. To answer the question of whether MuSIC barcodes could be constructed, characterized, and then reliably identified in single cells, we targeted a simple application consisting of two fluorescent protein (fp) barcodes. While this application is a small fraction of the theoretical potential, we reasoned it nevertheless provides a sizable library diversity for some biological applications and would support larger scale efforts if successful.

**Figure 1.**
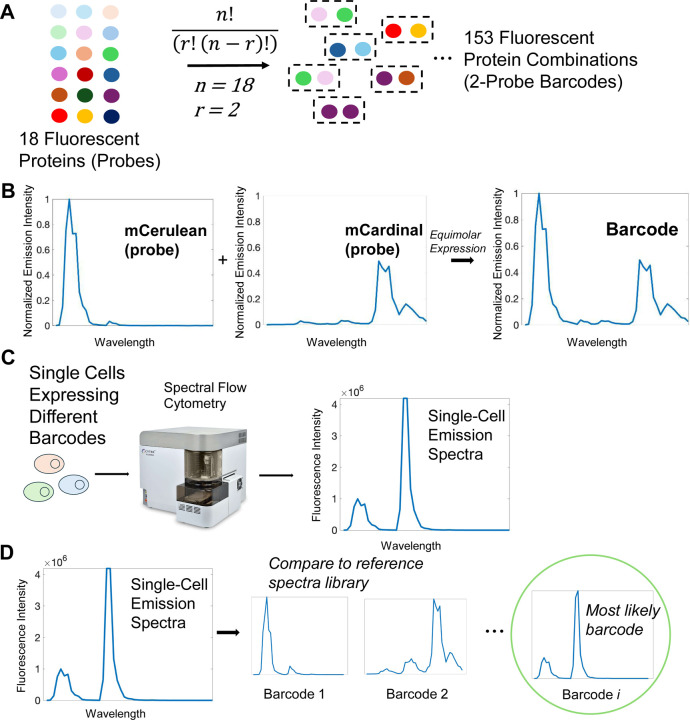
Barcodes and Their Analysis. **(A)** 153, 2-probe barcodes can be generated from 18 fluorescent proteins (fps). Combinations (vs permutations) are relevant because fp order does not affect the barcode. **(B)** Barcodes have a unique emission spectra arising from the two fps. HEK293 cells were transfected and individual fp spectra measured by spectral flow cytometry. The barcode spectra is illustrative as the sum of the two fp spectra. **(C)** Spectral flow cytometry measures emission spectra of individual cells with potentially different barcodes. **(D)** The emission spectra for each cell can then be compared to a reference library (containing spectra for individual fps and each barcode) to identify which barcode the cell mostly likely possesses.

Specifically, we selected 18 fps spanning UV-to-IR spectral properties (EBFP2^41^, mTagBFP2^42^, mT-Sapphire^43^, mAmetrine^43^, mCerulean3^44^, LSSmOrange^45^, mBeRFP^46^, mTFP1^47^, EGFP^48^, CyOFP1^49^, mClover3^50^, mVenus^51^, mPapaya^52^, mOrange2^53^, mRuby3^50^, mKate2^54^, mCardinal^55^, miRFP670^56^). These are called “probes”, in this case simply individual fps (Fig. 1A). A MuSIC barcode, in this study, is a combination of two probes. Given 18 fps and barcodes made of 2 probes, 153 unique barcodes can be generated. It is important to note here we only count combinations, where order does not matter, as opposed to permutations, where order matters. That is because in the targeted application using single-cell fluorescence emission spectra measurements, the order of the barcode would not be distinguishable. Furthermore, we consider combinations without replacement, that is, we do not consider a barcode consisting of two of the same fp. While these may exist in the eventual construction, we suspect inclusion of such barcodes will deteriorate detection reliability. That is because if only a single fp is identified, that may be due to a false negative for the 2^nd^ fp.

As an example, consider a cell expressing an mCerulean3 and mCardinal barcode (Fig. 1B). The individual fp spectra are invariant to whether a barcode is mCerulean3 / mCardinal or mCardinal / mCerulean3. The combination of these individual spectra gives the barcode spectra, balanced by their relative expression levels (in this example equimolar), which in principle are unique from all the other barcodes. This uniqueness, of course, depends on the spectral emission detector properties. If different single cells in a population are expressing different barcodes, full spectrum flow cytometry can be used to read out single-cell fluorescence emission spectra (Fig. 1C). These measured emission spectra can be compared to known references, from which the most likely barcode can be identified (Fig. 1D). This conceptual setup forms the basis of the experimental testing of the approach in what follows.

### Construction of a MuSIC Barcode Library

**Figure 2.**
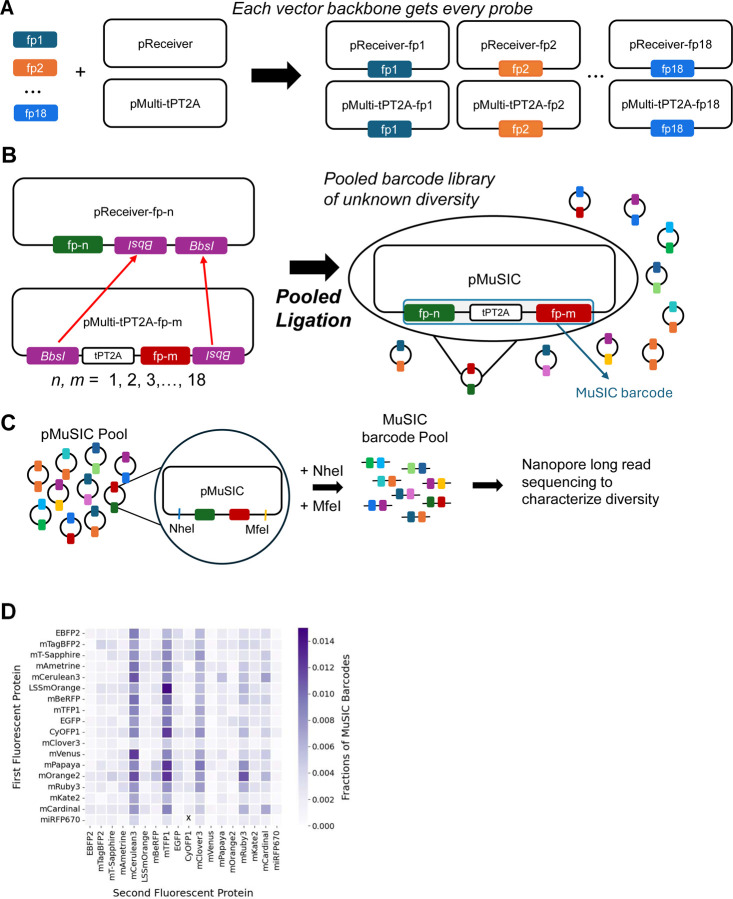
Barcode Library Construction and Validation. **(A)** Each of the 18 fluorescent proteins (fps) were cloned into the pReceiver and pMulti backbones. **(B)** The fps from pMulti were inserted into the pReceiver backbone by GoldenGate assembly in a pooled format to generate a pMuSIC barcode library. **(C)** The pMuSICs were double-digested with *NheI* and *MfeI* and then sequenced using a nanopore long read sequencer (MK-1C). This avoids PCR and short-read alignment which may be problematic due to fp homology. **(D)** Nanopore sequencing data from the pMuSIC pool were analyzed and individual fps in each barcode assigned and counted. Heatmap color denotes the percentage of reads attributed to a particular fp in either the 1st or 2nd position of the barcode. We verified that all potential barcodes are represented in the pool, except for miRFP670-CyOFP1, as indicated by the marked ‘x’. However, this barcode was later confirmed by sequencing to be present in the single MuSIC barcodes isolated for flow cytometry, specifically as pMuSIC-S94 (Table S16).

As mentioned above, MuSIC barcodes are constructed here by combining two fluorescent proteins (although they could be larger). To accomplish this, we generated two separate sets of probes (Fig. 2A). Each set has it’s own backbone, either pReceiver (pR) or pMulti (pM). Every fluorescent protein was cloned into each backbone. To subsequently generate barcodes, we took a pooled construction approach, via Golden Gate assembly with BbsI (Fig. 2B). For efficiency of design, barcode expression is driven by a single promoter in pReceiver, and the barcode elements are separated by 2A peptide sequences. We found that only a single 2A sequence led to some fusion protein generation (which would generate “confusing” spectra), and that a tandem 2A sequence^57^ was necessary to make such fusion events largely negligible (Fig. S1). Each of the 18 plasmids in both sets were pooled and then digested. The digested pR and pM pools were combined in equimolar amounts and a ligation was performed forming a pooled barcode library. We note that this approach is designed to scale to larger barcodes but for proof-of-concept purposes here we analyze barcodes with two fluorescent proteins.

The proportion of each barcode type in the pooled library was unknown, so we performed sequencing (Fig. 2C). When considering the type of sequencing to use, we had to address two main concerns. One was that we had to know the correct pairing of the fluorescent proteins of each barcode. Short read sequencing may not provide enough data to determine such pairing, so we decided to use long read sequencing. The next concern was that some fluorescent proteins have high sequence homology, which can complicate PCR^58,59^. Nanopore sequencing became apparent as the best option for our purpose as it would allow us to perform long read sequencing while also eliminating the need for PCR that is required by most other deep sequencing technologies. To analyze the sequencing results, we determined which fluorescent protein was in the first position, and which was in the second (see Methods and Code). While there appears to be bias towards mCerulean3, mTFP1, and mClover3 in the second position, observable due to the deeper color in those columns (possibly due to systematic pipetting error occurring during the pooling/digestion stage), all barcode combinations (with the exception of one subsequently found during single colony isolation below) were found to be present, as indicated by color spread throughout the heatmap visualization (Fig. 2D). These results validate the cloning process used to generate the MuSIC barcode pool and provide a plasmid library ready for testing the approach in cells.

### Unmixing of Single Fluorescent Proteins and Generating Reference Spectra

**Figure 3.**
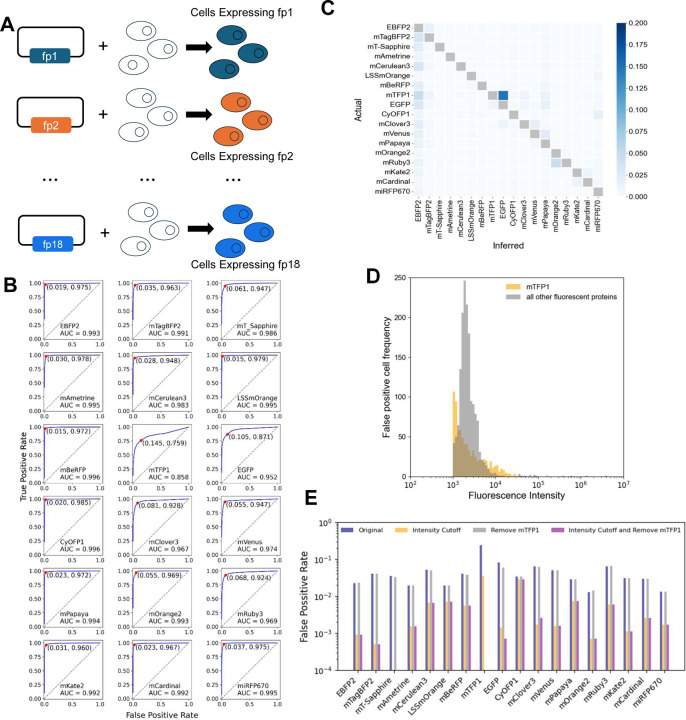
Single-Cell Identification of Individual Fluorescent Proteins. **(A)** Plasmids containing single fluorescent proteins (fps) are transfected into HEK293 cells and assessed by spectral flow cytometry. **(B)** The data from A were subjected to unmixing, thresholding, and classification to generate a receiver-operator characteristic curve for each fp. Area under the curve is shown in each inset, along with optimal threshold point. **(C)** False positive rate at an optimally chosen classification threshold for each fp. Actual is the transfected fp; inferred is the incorrectly identified fp. **(D)** The number of cells misclassified as a function of their maximum fluorescence intensity. Cells with low intensity are predominantly the misclassified ones, and mTFP1-containing cells are the most frequently misidentified. **(E)** False positive rates for each fp based on different modifications to the data set as indicated. Note the log y-axis. Cells were eliminated from consideration by an intensity cutoff (10^4^), mTFP1 was removed from the reference matrix, or both.

Before testing the identification of barcodes, we wanted to ensure that each of the 18 fluorescent proteins were deconvolvable from one another, while also generating the reference spectra for each fp needed for the identification of barcodes. To test whether the 18 fps could be unmixed, we individually transfected each of the 18 pR plasmids (Fig. 3A) and assayed cells with a spectral flow cytometer. This experiment was performed at least twice independently to test whether the emission spectra were stable and reproducible, with results showing near uniform concordance (Fig. S2).

To evaluate performance, we generated a ROC curve for each of the 18 fps, by sliding a classification threshold applied to the fp abundance estimated from spectral unmixing (Fig. 3B). In general, fps could be identified with high fidelity, with area under the curve (AUC) close to 1 for many, with the clear exception of mTFP1 performing much worse. To better understand the poor performance of mTFP1, as well as unmixing errors, we analyzed the false positive rates for each fp in each of the 18 samples (Fig. 3C). The false positive rates are calculated using an optimum classification threshold, which corresponds to the point closest to 100% TPR and 0% FPR on the ROC curve (indicated in Fig. 3B). EGFP and mTFP1 were often misclassified for one another, whereas most other errors do not show a significant pattern.

We reasoned that low fluorescence intensity of a single cell may also be a factor in misclassification. The distribution of false positive classification frequency as a function of signal intensity demonstrates that cells with intensity lower than 10^4^ (relative intensity) are responsible for most errors, and that again mTFP1 is responsible for most errors (Fig. 3D). Removing cells with such low intensity eliminated a substantial amount of false positive errors (Fig. 3E). Removing mTFP1 as a classification possibility had a slight to no impact on most other fps, but of course did remove by far the worst performing fp (mTFP1) (Fig. 3E). We conclude that individual fps can be reliably classified, such that moving forward mTFP1 containing barcodes will not be considered, and a single-cell fluorescence intensity cutoff will be applied (~10^4^) to improve classification performance. This reduces the total number of possible barcodes to 136 for further analysis.

### Identification of MuSIC Barcodes in Single Cells

**Figure 4.**
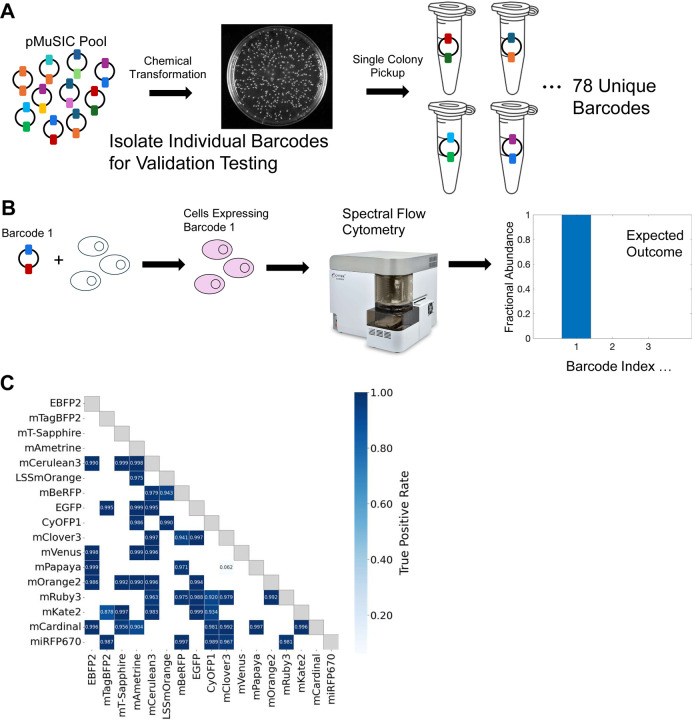
Single-Cell Identification of pMuSIC Barcodes. **(A)** Single-colony pickup after pool transformation enables isolation of individual barcode plasmids. **(B)** Single pMuSIC barcodes are transfected and then analyzed to assess barcode identification performance. **(C)** True positive rates for barcodes isolated and assayed as in A-B. Here, the intensity cutoff was applied, and mTFP1-containing barcodes were removed from the analysis as in Fig. 3. White squares denote barcodes that were not isolated in experiments from A.

To assess the validity of MuSIC barcode identification in single cells, we decided to isolate individual barcodes so precisely controlled experiments could be performed (Fig. 4A). To do this, we performed repeated single-colony pickup following transformation of the pooled library, and then determined which barcodes were found by sequencing (Fig. S3). From 114 colonies we obtained 112 pMuSIC plasmids, of which 108 were identified as valid MuSIC barcodes, while 5 with identical fluorescent proteins (e.g. EGFP-EGFP) were classified as invalid. Out of these, 69 unique MuSIC barcodes were chosen to diversify across the 18 fps at each probe position. As noted above, we did not further analyze any mTFP1 containing barcodes, which left 55 unique barcodes for analysis. We transfected cells with single MuSIC barcodes and analyzed them with full spectrum flow cytometry (Fig. 4B). Overall, barcodes could be identified with high fidelity (Fig. 4C). One exception was the combination of mClover3 and mPapaya, which was incorrectly identified in nearly every cell. Other barcodes that had moderate but not excellent classification performance were mTagBFP2/mKate2, mAmetrine/mCardinal, CyOFP1/mKate2, and CyOFP1/mRuby3. The spectra of the individual fluorescent proteins and of the expected versus measured barcodes (Fig. S4) reveal a possible shared explanation for the reduced performance of these barcodes. In theory, each of these barcodes would be uniquely resolvable, based on expected (ideal) spectra (Fig. S4). However, the expression of the first fluorescent protein in the barcode is much lower than expected, which follows from the difference between the expected (ideal) spectra and the observed spectra essentially missing the first fluorescent protein characteristics (Fig. S4). This gives rise to an observed spectra from which it is difficult to infer the correct barcode, since the spectra looks essentially only like the second fluorescent protein. Why only this handful of barcodes display this issue remains unclear, but it may be due to peptide scars following tPT2A cleavage leading to reduced expression of the first fp in the barcode. We conclude that overall, MuSIC barcodes can be identified with excellent performance in cells using spectral flow cytometry, with a few exceptions.

## Discussion

Cell barcoding is a core technique in the biological sciences. Barcodes are typically made from either nucleic acids or fluorophores. Ideally, the barcoding method would enable (i) a large number of unique barcodes to be constructed (i.e. library size or diversity) such that many single clones or populations could be analyzed simultaneously, (ii) non-destructive readout to enable tracking the same cells or population over time, and (iii) fast and/or inexpensive readout to enable the same but additionally measurement across many conditions. Historically, nucleic acid methods excel at (i) but struggle with (ii) and (iii), whereas fluorescence methods, the opposite. In this work, we establish a proof-of-concept that combinations of fluorophores and spectral emission measurements increase potential library size for fluorescence-based measurements. We create a library of 153 barcodes from 2-way combinations of 18 fluorescent proteins, and demonstrate that many can be reliably identified with true positive rates of >99% using spectral flow cytometry.

Because this library is genetically-encoded, it could potentially be coupled to small gRNA libraries for fast single-cell genetic or genetic interaction screening, or for lineage tracing of hundreds of clones. In terms of genetic interaction screening, current methods based on incorporation of two guide RNAs in a single plasmid^24,26^ are fundamentally limited by the number of individual bacteria that can contain a unique plasmid in the library, whereas our approach may not be so limited. Spectral live-cell microscopy imaging to trace clones in space and time could enable analysis of an even greater number of clones, since the likelihood of observing the same barcode in the same spot is lower, and thus the same barcode may be repeated in different spatial locations given appropriate experimental design.

The established library size here, while somewhat greater than the potential of current fluorescent protein approaches^4,35,36^, remains far below that possible using nucleic acids^25,26,28–34^. We envision multiple routes to higher library diversity. One could be simply use more advanced demultiplexing and classification algorithms, which could allow for more accurate detection of fluorescent proteins and larger barcodes. Here, we employed a somewhat simple approach based on classical linear unmixing (non-negative least squares), followed by selection of the two fluorescent proteins most likely to be present. The continuous estimated abundance from linear unmixing was converted to a ratio score by comparison to a threshold established from analysis of single fluorescent proteins. The two fluorescent proteins (because it is a two-member barcode) with the highest scores were then said to comprise a cell’s inferred barcode, thus binarizing the output for casting as a classification problem. We expect modern machine learning methods could further improve the already good classification performance we observed^60,61^.

While differences in classification methods could impact effective library size and performance, the largest potential increases come from modifying the number of fluorescent proteins, and the number of fluorescent proteins per barcode. This proof-of-concept work is based our recent theory and simulations^38^, that considers not only individual fluorescent proteins, but fluorescent protein fusions as independent potential barcode members. The approach, called multiplexing using spectral imaging and combinatorics (MuSIC)^39^, works because when a fluorescent protein fusion is constructed, if substantial FRET occurs from one to the other, then the emission spectra of the fusion protein cannot be constructed from a linear combination of the two individual fluorescent proteins, and is thus unique in terms of linear unmixing. Based on the same 18 fluorescent proteins used here, we expect that hundreds of so-called MuSIC probes could be constructed using 2 and 3-way fusion proteins, which has some technical precedent^62–64^. Simulations matched to current full spectrum flow cytometry equipment suggested that using only 2-probe barcodes, from a pool of hundreds of barcodes, could enable human genome scale library diversity (~10^4^ – 10^5^)^38^. If one could make 3-probe barcodes from 200 probes, the potential diversity becomes ~10^6^. Future work will explore these avenues.

Another potential improvement relates to hardware. Emission spectra scanning has historically been possible but technically far more challenging than simple filter-based four color measurements. However, full spectrum flow cytometers have recently enabled much greater multiplexing than previously possible^65,66^. White lasers that enable excitation wavelength tuning are now more common^67^, as are microscopes with spectral detection^68,69^. All such hardware advances are expected to enhance feasibility of reading out these emission spectral barcodes and to increase the fidelity of classification for larger libraries.

There are notable limitations of the approach which derive from sequence homology among many fluorescent proteins. First is how to stably deliver such barcodes to cells. Many barcoding approaches use pooled lentiviral libraries, but creating lentiviral libraries with fluorescent protein combinations is likely to be problematic due to template switching from sequence homology^30,32^. Silent mutations (and perhaps other non-critical non-synonymous mutations) could be used to potentially overcome such issues^70^, although such variants would need careful testing because silent mutations can affect expression through mRNA folding^71^. Delivery to cells may be enabled by simple transfection coupled with recombination-based landing pad systems for control of single copy integration in safe harbors through selection and counter-selection^72^. Barcode library construction may be hampered by difficulty performing PCR on segments and constructs containing large homologous regions from fluorescent proteins. Indeed, here we avoided PCR of intact barcodes because of such concerns, which led us to nanopore sequencing-based characterization of the pooled barcode library. Although recombinase-deficient *E. coli* strains are routinely used, recombination of barcodes may be a problem during library propagation or in the eventual target cells-of-interest. However, the success of Brainbow systems *in vivo* argues this may achievable^4,35,36^. As the size of probes and barcodes grow, the limits of plasmid length may be reached. Lastly, we removed mTFP1 from analysis here as our particular 4-laser spectral flow cytometer struggled to accurately demultiplex it. However, we suspect a 5-laser model may be successful for such purposes, and clearly the particulars of which fluorescent proteins can be used is highly dependent upon the excitation channels used and the emission spectra detection properties.

In conclusion, we demonstrate here a library of ~150 fluorescence barcodes can be constructed from combinations of 18 fluorescent proteins and accurately classified using spectral flow cytometry. There is substantial room for growth in library size via both design and hardware. We expect that this approach could fulfill a large gap in barcoding technology to provide a large diversity library with fast, inexpensive and non-destructive readout. This could enable large-scale, single-cell genetic and genetic interaction screening or lineage tracing applications.

## Methods

### General Experimental Procedures

#### Workflow Overview

All products from restriction enzyme digestion, ligation, Gibson Assembly, and Goldengate cloning were directly introduced into either NEB 5-alpha Competent *E. coli* (*New England Biolabs*, Cat# C2987I) or DH5α homemade chemically competent cells via transformation as described below. Single colonies were inoculated into 2.5mL LB medium for overnight culturing. Candidates were screened by colony PCR. Plasmids were purified by the PureYield miniprep system (*Promega,* Cat# A1222) and confirmed by double digestion. All final plasmids were verified by sequencing (*Genewiz* and *Plasmidsaurus*). They are available on Addgene (pool—84895; individual plasmids—85024).

#### Preparation of Chemically Competent Cells

One microliter of DH5α competent seeds from NEB 5-alpha Competent *E. coli* (*New England Biolabs*, Cat# C2987I) was inoculated in 2.5mL SOB medium (*Fisher BioReagents*, Cat# BP9737-500) in a 14mL round-bottom test tube (*Corning*, *Falcon Cat#* 352059), incubated at 225rpm and 37°C overnight. The overnight culture was then diluted 1:1,000 (v/v) into 200mL SOB medium and incubated at 37°C at 225rpm until the OD_600_ reached 0.32 (about 5 hours). After chilling on ice for 10 minutes, cells were centrifuged at 1000*g* (~3000rpm) for 10 minutes at 4°C with a Fiberlite F15-8×50c rotor (*Thermo Scientific*) in a Sorvall Legend XFR refrigerated centrifuge (*Thermo Scientific*) and gently resuspended in 16mL ice-cold CCDB80 transformation buffer. This buffer was prepared using 10mM potassium acetate (*VWR Chemicals BDH*, Cat# 9254-500G), 80mM calcium chloride dihydrate (*Sigma-Aldrich*, Cat# C3306-100G), 20mM manganese chloride tetrahydrate (*Alfa Aesar*, Cat# 44442-25G), 10mM magnesium chloride hexahydrate (S*igma-Aldrich*, Cat# 13152-1KG), 10% glycerol v/v (*ICN Biomedicals*, Cat# 800687), and pH6.4 adjusted by 0.1N hydrochloric acid (*VWR Chemicals BDH,* Cat# BDH3028-2.5LG). After chilling the cells on ice for another 20 minutes, they were centrifuged again at 1000*g* for 10 minutes at 4°C. The cell pellet was gently resuspended in sterile ice-cold CCMB80 buffer to achieve a final OD_600_ of ~1. Cells were aliquoted (50 μL) into pre-chilled sterile microcentrifuge tubes and frozen in a dry ice/ethanol bath for 5 minutes before long-term storage at −80°C. Transformation efficiency with pUC19 Control DNA at different dilution ratios *(New England Biolabs,* Cat# N3041A*)* showed ~7.4 × 10^7^ cfu/μg DNA.

#### Chemical Transformation

A 50μL aliquot (from above) of lab-prepared DH5α competent cells were thawed on ice for 5–10 minutes. One microliter (unless otherwise noted) of ligation (or other) product was added to the DH5α cells, and the tube was carefully flicked 5 times to mix the DNA and competent cells. After 30 minutes incubation on ice, the cells were heat-shocked at 42°C for exactly 30 seconds, followed by immediate transfer back to ice for 5 minutes. 950μL of room temperature SOC outgrowth medium (*New England Biolabs, Cat#* B9020S) was added to the cells in a 5 mL polypropylene round-bottom tube (*Corning*, *Falcon Cat#* 352063). The mixture was shaken at 225rpm at 37°C for 1h and around 100μL was then spread onto a selection LB agar plate. The plates (*Fisher Scientific*, Cat# FB0875712) were made from LB Agar Lennox (*Fisher BioReagents*, Cat# BP9745-500) according to the manufacturer’s instruction and supplemented with 50μg/mL Kanamycin (*VWR*, Cat# 0408-10G) for pReceiver(pR) backbone or 25μg/mL Chloramphenicol (*ACROS Organics*, Cat# AC227920250) for pMulti (pM) backbone.

#### Inoculation

Around 2.5μL glycerol stock or a single colony was inoculated into 2.5mL autoclaved LB medium in a 14mL round-bottom test tube (*Corning*, *Falcon* Cat# 352059), incubating at 225rpm, 37°C overnight. The recipe for preparing LB medium is as follows: 5g Tryptone (*Fisher Bioreagents*, Cat# BP1421-500), 2.5g Yeast Extract, Bacteriological Grade (*VWR Life Science*, Cat# J850-500), 5g Sodium Chloride (*Fisher BioReagents*, Cat# BP358-1). Adjust the pH to 7.0 with 10N Sodium Hydroxide Solution (*Fisher Chemical*, Cat# SS255-1) and add Milli-Q water (Millipore Advantage A10 water purification system) to a final volume of 500mL.

#### Touchdown Colony Polymerase Chain Reaction (PCR)

Single colonies were screened by touchdown colony PCR with screening primers (*Integrated DNA Technologies, IDT*) as shown in Table S1. For each PCR reaction, approximately 0.2μL of overnight culture was added to the reaction mixture containing 1.5μL 10x *Taq* ThermoPol^®^ buffer (*New England Biolabs*, Cat# B9004S), 0.3μL 10mM dNTP mix (*Thermo Scientific*, Cat# R0192), 0.2μL of 20μM forward primer, 0.2μL of 20μM reverse primer, 0.1μL *Taq* DNA polymerase (*New England Biolabs*, Cat# M0267L), and autoclaved Milli-Q water to a final volume of 15 μL.

The PCR was initiated at 95°C for 5 minutes to release the DNA. A series of 10 touchdown cycles were then done, consisting of 95°C for 30 sec, 65-53°C decreasing 1°C per cycle for 30 sec, and 68°C for 30 sec. An additional 25 PCR cycles with an annealing temperature at 52°C was performed, followed by a final extension at 68°C for 5 minutes. Candidates were selected by performing electrophoresis on a 1% (w/v) agarose gel (*Fisher BioReagents*, Cat# BP160-500) prepared in 1x TAE buffer, which was diluted from 50x TAE buffer. The recipe for preparing 50x TAE buffer is as follows: 121g Tris (*Thermo Scientific*, Cat# J22675-A1), 28.55mL Glacial Acetic acid (*VWR BDH Chemicals*, Cat# BDH3098-3.8LP), and 50mL 0.5M Ethylenediaminetetraacetic acid (*Alfa Aesar*, Cat# A10713), pH 8.0. Add Milli-Q water to a final volume of 500mL.

#### Restriction Digest

##### Digestion for Cloning Purposes

Purified DNA fragments from PCR products or plasmid DNA were subjected to double restriction enzyme digestion to facilitate downstream applications such as DNA ligation, Gibson assembly, Golden Gate cloning, or Nanopore sequencing. For each reaction, approximately 1 μg of plasmid DNA was added to a digestion mixture containing 5 μL of rCutSmart Buffer (New England Biolabs, Cat# B6004S), 10–20 units of each restriction enzyme, and autoclaved Milli-Q water, bringing the final volume to 50 μL. The reaction mixture was incubated at 37°C overnight to achieve maximum digestion efficiency.

##### Digestion for Verification Purposes

Candidates from colony PCR screening were confirmed by single linearization or double digestion with proper restriction enzymes as shown in Table S2. For each reaction, approximately 0.5μg plasmid DNA was added into the digestion mixture containing 1.5μL rCutSmart^™^ Buffer (*New England Biolabs*, Cat# B6004S), 10 units of each restriction enzyme, and autoclaved Milli-Q water to a final volume of 15 μL. For verifying the presence of the target band, the reaction mixture was incubated at 37°C for at least 4 hours to achieve sufficient digestion efficiency.

### Molecular Cloning

#### Single Plasmid Construct

##### Construction of pReceiver(pR) and pMulti(pM) Backbones

pR, pR-NheI, pM, and pM-tPT2A backbones are shown in Fig. S5. pR and pM variants were designed to contain different antibiotic resistance genes. All sequences of synthetic DNA fragments used for construction of these backbones are listed in Table S3. All PCR was performed using the primers listed in Table S4 and Q5 High-Fidelity DNA Polymerases (*New England Biolabs*, Cat# M0491L), following the manufacturer’s instructions.

For the pR backbone, the plasmid was divided into three parts: the f1 origin (f1ori), Kanamycin resistance cassette, and the CMV promoter. The f1ori was synthesized by *IDT* and then further amplified. The other two parts were similarly amplified from mTFP1-N1 (*Addgene*, #54521). Amplicons from synthesized DNA fragments were purified using the Monarch PCR & DNA Cleanup Kit (New England Biolabs, Cat# T1030L), while amplicons from plasmids were purified using the Monarch Gel DNA Extraction Kit (New England Biolabs, Cat# T1020L) to eliminate any remaining background. The pR plasmid was then created with these purified PCR products using Gibson Assembly Master Mix (*New England Biolabs*, Cat# E2611L) with a picomolar ratio as 1:1:1 and incubated at 50°C for 1h.

For the pR-NheI backbone, an NheI restriction enzyme cutting site was inserted before the CMV enhancer by overlap extension PCR of the SnaBI-ApaLI double-digested fragment to create the SnaBI-*NheI*-ApaLI insert, which was then loaded onto the pR backbones through SnaBI (*New England Biolabs,* Cat# R0130S) and ApaLI (*New England Biolabs,* Cat# R0507S) restriction enzyme cutting sites followed by ligation with *T4* ligase (*New England Biolabs,* Cat# M0202L) to create pR-NheI backbones for further nanopore sequencing.

For the pM backbone, the plasmid was also divided into three parts: the T2A-f1ori, Chloramphenicol (CMR) resistance cassette, and the CMV promoter. The T2A-f1ori and CMR cassettes were synthesized by *IDT* and *Genewiz*, respectively. The CMV cassette was amplified and purified as above. The pM plasmid, designed to contain the *BbsI-T2A-BsaI-spacer-BsaI-BbsI* cassette, was constructed using these purified PCR products through Gibson assembly as described above.

For the pM-tPT2A backbone, a tandem P2A-T2A(tPT2A) DNA fragment was synthesized by *IDT* and then amplified and purified as described above. Both purified tPT2A amplicons and the pM backbone plasmids were treated with BbsI-HF (*New England Biolabs*, Cat# R3539L) to create the insert and the vector, respectively. They were purified with a Select-a-Size DNA Clean & Concentrator kit (*Zymo Research*, Cat# D4080) and then ligated by Goldengate assembly with an insert-to-vector ratio as 3:1 and the *T4* ligase (*New England Biolabs,* Cat# M0202L), following the manufacturer’s instructions.

##### Cloning Individual Fluorescent Proteins into Different Backbones

The workflow diagram for the molecular cloning design to insert individual fluorescent proteins into pR, pR-NheI, pM, and pM-tPT2A backbones is illustrated in the gray background box in Fig. S6A. Sequences of fluorescent proteins, listed in Table S5, were designed to avoid recognition sites of type IIS restriction enzymes, including BsaI, Esp3I, and BbsI, through the implementation of silent mutations. They were amplified with Q5 High-Fidelity DNA Polymerases (*New England Biolabs*, Cat# M0491L), using primers according to Fig. S6B and S6C and Table S6.

For those reverse primers longer than 60nt, as shown in pR-fp-reverse (3’−5’) in Fig. S6B, each reverse primer was split into two shorter ones. The first sub-primer ended at the GAAG sequence within the second BbsI recognition site, just after the ATAATT cassette containing stop codon (TAA), while the second sub-primer began at the CTCC sequence within the first BbsI cutting site, directly following the fluorescent protein. This design allowed for a 22bp overlap between the two short sub-primers. After two rounds of PCR, the final amplicons containing the BsaI-fp-TAA-BsaI cassette were prepared as inserts. They were then purified and subsequently inserted into pR or pR-NheI backbones through BsaI sites using Goldengate assembly with *T4* ligase (*New England Biolabs,* Cat# M0202L) and BsaI-HF (*New England Biolabs*, Cat# R3733L).

Those inserts for pM backbones were amplified directly and inserted into pM and pM-tPT2A backbone to generate different fluorescent probes, such as pM-fp and pM-tPT2A-fp.

##### Cloning a Fusion Fluorescent Protein into pMulti Backbone

To compare the cleavage efficiency of T2A versus tPT2A in MuSIC barcodes, we cloned the mTFP1-mVenus fusion into pM backbone to create a positive-control probe for poorly cleaved MuSIC barcodes. The mTFP1 and mVenus were linked with seven amino acids (AGGGGLG), as described previously^39^.

The pM backbone was treated with BsaI-HF (*New England Biolabs*, Cat# R3733L) overnight to prepare the vector. At the same time, mTFP1 was amplified with primers as listed in Table S7 to generate the insert (mTFP1-Esp3I-TAA-Esp3I), allowing the mVenus to be inserted through the Esp3I sites. Both the first insert and the vector were purified and ligated by Goldengate assembly with the BsaI-HF (*New England Biolabs*, Cat# R3733L) and *T4* ligase (*New England Biolabs,* Cat# M0202L), following the manufacturer’s instructions.

The resulting plasmid (pM-mTFP1-Esp3I-TAA-Esp3I) was screened and verified through the general procedures described above. This plasmid was then used as the vector for the insertion of a second fragment (linker-mVenus), which was amplified from the pQLinkHD-mVenus plasmid (*Addgene*, #118861)^39^. Both the second insert and vector were purified and ligated by Goldengate assembly with Esp3I (*New England Biolabs*, Cat# R0734L) and *T4* ligase (*New England Biolabs,* Cat# M0202L), following the manufacturer’s instructions, to generate the pM-mTFP1-linker-mVenus plasmid as the control probe.

##### Construction of MuSIC Barcodes (pMuSICs) for 2A cleavage efficiency comparison

Both pR-fps and pM-fps were treated overnight with BbsI-HF (*New England Biolabs*, Cat# R3539L). The digestion products from the pR probes were purified with the Monarch PCR & DNA Cleanup Kit (New England Biolabs, Cat# T1030L) to serve as the vector, while those from pM probes (T2A-fp) were isolated using the DNA Gel Extraction Kit (New England Biolabs, Cat# T1020L) to serve as the insert. The purified vector and insert were then mixed at a molar ratio of 1:5 (vector to insert) and combined by Goldengate assembly through BbsI cutting sites to generate pR-fp-T2A-fp as the pMuSIC-T2A constructs for cleavage efficiency comparison (Fig. S6A).

Similarly, pR-NheI-fp and pM-tPT2A-fp were digested and purified prior to Goldengate assembly. These were then used to create pR-NheI-fp-tPT2A-fp as the pMuSIC-tPT2A constructs for cleavage efficiency comparison (Fig. S6A).

#### pMuSIC v1.0 Library Construction

##### Construction of MuSIC-barcode pool

First, 0.45 pmol of each pR-NheI-fp and pM-tPT2A-fp were pooled to create two separate pR and pM probe libraries, respectively. Each of these libraries were then digested separately overnight using BbsI. The pR probe pool was purified using a Monarch PCR & DNA cleanup kit, while the digested pM probe pool was purified using the DNA Gel Extraction Kit (New England Biolabs, Cat# T1020L) to isolate the insert fragment (tPT2A-fp). To prevent self-ligation, the pR probe pool was treated with Quick CIP (*New England Biolabs,* Cat# M0525S) and purified using a DNA cleanup kit as described above. The pR and pM probe pools were assembled at a 1:5 vector-to-insert molar ratio using *T4* ligase (*New England Biolabs,* Cat# M0202L) and BbsI-HF (*New England Biolabs*, Cat# R3539L), creating the MuSIC-Barcode sample group. A ligation reaction without the insert (pM probe pool) was also set up as the negative control group (Fig. S3), which was verified to have negligible colonies post-transformation.

##### Pool Chemical Transformation

The assembly products from both groups were diluted at 1:10 and 1:100 ratios with autoclaved Milli-Q water (Fig. S3). One microliter of each diluted assembly product was used to transform DH5α chemically competent cells (made as above). For each dilution in each group, 100μL of the transformation culture were spread onto an LB agar/Kana+ plate A while the rest were onto a second plate B. After incubation for 21 hours at 35°C, the colonies were imaged using a ChemiDoc^™^ XRS+ imager (*Bio-Rad*). A total of 4461 positive colonies were counted in the sample group, while only 1 colony was found in the negative control group.

##### Isolation of Single MuSIC Barcodes

Approximately 114 single colonies on plate A of 1:100 dilution of sample pool were selected as the single positive controls for subsequent spectral unmixing (Fig. S3). Among the first 24 colonies screened by colony PCR, only one was verified to be negative. The plasmid DNA from the rest of the candidates was extracted using a PureYield^™^ miniprep system (*Promega,* Cat# A1222) and further confirmed by double digestion, as described in General Experimental Procedures. With this high positive rate, the remaining single colonies were directly subjected to miniprep. The identity of the barcode in each plasmid was verified by sequencing (*Plasmidsaurus*). Among the 113 plasmids sequenced, one was confirmed to be negative, five were verified to contain identical fps, and the remaining 108 were verified to contain valid MuSIC barcodes.

##### Pooled MuSIC-barcode Library Generation

Transformed colonies were scraped from the agar plate of the sample group into 10 mL of LB medium supplemented with 50 μg/mL kanamycin (*VWR*, Cat# 0408-10G) in a sterile 50 mL polypropylene conical tube (*Corning*, *Falcon* Cat# 352070) using a cell scraper (*Fisher Scientific*, Cat# 08100-241). The cell scraper was washed with 4 mL of LB/Kana+ twice, while the agar plate was washed with 2 mL of LB/Kana+. The resulting cell/medium mixture was carefully transferred into the conical tube by pipetting to maximize the transfer of colonies into the tube. The cell/medium mixture was then added to 170mL of LB/Kana+ in a 1L flask and incubated at 250rpm at 37°C for 18 hours until O.D.600 reached 3, as measured by a NanoDrop^™^ 2000 spectrophotometer (*Thermo Scientific*). The pooled pMuSIC library was isolated by the PureYield^™^ plasmid maxiprep system (*Promega*, Cat# A2393).

### Cell Culture and Transfection

#### Cell Culture

HEK293T cells were obtained from ATCC (CRL-3216) and grown in Dulbecco’s Modified Eagle’s Medium (DMEM) (*Gibco*, Cat# 10313021) supplemented with 10% (v/v) fetal bovine serum (*Gibco*, Cat# 10082139) and 5% L-Glutamine (*Corning*, Cat# 25-005-CI) under a 5% CO_2_ atmosphere at 37°C. Cells were sub-cultured every 2-3 days to prevent confluence. This process involved lifting with 0.25% Trypsin (*Gibco*, Cat# 25200-072), followed by centrifugation at 100*g* (~1000rpm) for 5 minutes at room temperature, and a final resuspension in full growth medium (as described above).

#### Transfection

For flow cytometry experiments, HEK293T cells (6 × 10^4^) were seeded into 12-well plates (*Corning*, *Falcon* Cat# 353043) 24h before transfection. Once cells reached approximately 20% confluency, they were transfected with pR or pM or pMuSIC plasmids at concentrations ranging from 50ng to 300ng using *Lipofectamine 3000* (*Invitrogen,* Cat# L3000-008) according to the manufacturer’s instructions. For higher transfection efficiency, cell medium was replaced with DMEM/L-glutamine one hour before transfection and supplemented with 10% FBS four to six hours after transfection.

For western blotting, HEK293T cells (5 × 10^5^) were seeded into 6-well plates (*Corning*, *Falcon* Cat# 353046) and incubated for 24h to achieve ~50% confluency. Three micrograms of DNA were transfected into each well by Lipofectamine 3000 (*Thermo Fisher Scientific*, Cat# L3000001), with triplicates performed for each sample.

### Western Blotting

#### Sample Preparation and Quantification

Forty-eight hours post-transfection, cells were lysed in 100μL of the fresh-made ice-cold RIPA buffer per well^73^. The plates were kept on ice for 15 minutes and the cells were agitated every 5 minutes to ensure thorough lysis. The lysates were scraped off with cell scrapers (*Stellar Scientific*, Cat# TC-CS-25) and transferred into pre-chilled 1.5 mL microcentrifuge tubes. Each tube was vortexed for 5 sec and repeated 3 times to homogenize cell debris. Cells were then centrifuged at 4°C at approximately 16,000*g* for 5 minutes to clear the lysate from debris.

About 10μL supernatant was stored at −80°C or directly used for protein quantification with a Pierce Rapid Gold BCA Protein Assay kit (*Thermo Scientific*, Cat# A53225) according to the manufacturer’s instructions. The standard curve was generated using Bovine Serum Albumin Standard Ampules (*Thermo Scientific*, Cat# 23209), and the concentration of the samples was determined with a BioTek *SynergyH1* multimode microplate reader. On the other hand, approximately 270μL supernatant from each sample (triplicates) was transferred into a new pre-chilled 1.5 mL micro-centrifuge tube and mixed with 4x Laemmli sample buffer (*Bio-Rad*, Cat# 161-0747) that had been supplemented with 10% 2-Mercaptoethanol (*Fisher Scientific*, Cat# O3446I-100) for sample preparation. The tubes were then heated at 95°C for 5 minutes in a dry block heater and stored at −80°C for long-term storage.

#### Immunoblotting

Thirty micrograms of protein samples were loaded into wells along with a Chameleon Duo Pre-Stained Protein Ladder (*LICOR*, Cat# 928-60000). The proteins were separated by a Tris (*Thermo Scientific*, Cat# J22675-A1) - glycine (*Thermo Scientific*, Cat# A13816.0E) SDS gel, with all ingredients listed in Table S8 in the Mini PROTEAN Tetra Cell (*Bio-Rad*, Cat# 165-8001). The protein samples were subsequently transferred onto a 0.2μm Immun-Blot PVDF Membrane (*Bio-Rad*, Cat# 162-0177) in a glycine/methanol (*Fisher Chemical*, Cat# A452SK-4) transfer buffer at 100V for 1 hour. The membrane was blocked with 0.1% (v/v) Tween-20 (*Fisher Scientific*, Cat# BP337-100) in Tris-buffered saline (TBST) supplemented with a final concentration of 5% BSA w/v (*Fisher Scientific*, Cat# BP1600-100) for 1 h at room temperature and then incubated with the primary antibody solution with gentle rocking on a BioRocker 2D rocker (*Denville Scientific INC*) at 4°C overnight. After washing three-times with TBST buffer at room temperature, 15 minutes each, the membrane was incubated with the secondary antibody solution while gently rocking on a rotator mixer stirrer (*Fisher Scientific 2309FS*) at room temperature for 30 minutes. The membrane was washed again as previously described (but without Tween) and the target protein bands were visualized by a LI-COR Odyssey infrared fluorescence scanning system.

Immunoblotting was performed using antibodies diluted as specified in Table S9. All primary antibodies were diluted as above in TBST supplemented with a final concentration of 5% BSA w/v and 0.02% Sodium Azide w/v (*Thermo Scientific Chemicals,* Cat# 190380050) while the secondary antibodies were diluted in 1x TBST buffer supplemented with a final concentration of 0.1% SDS (*Fisher Bioreagents*, Cat# BP2436-1) and 0.02% Sodium Azide w/v.

### Flow Cytometry

#### Sample Preparation

Forty-eight hours post-transfection, HEK293T cells were trypsinized using 0.25% Trypsin (*Gibco*, Cat# 25200-072) and centrifuged at 300*g* for 5 minutes at 4°C. Cell pellets were then washed with 1 mL of pre-chilled phosphate-buffered saline (PBS) (*Fisher BioReagents*, Cat# BP2944-100) and centrifuged as above. The cells were resuspended in 0.5ml ice-cold FACS buffer (PBS, 1% BSA w/v) for analysis using a *Cytek Aurora* spectral flow cytometer. Cells transfected with pR backbone were used as a negative control.

#### Acquisition and Analysis

Cells were gated based on FSC and SSC, and singlets were gated by FSC-H versus FSC-A. Cytek Aurora settings are initially configured for the use of SpectroFlo QC Beads (*Cytek*, Cat# B7-10001), which are ~3 μm in diameter. These settings are not optimal for analyzing HEK293T cells, which are 11 to 15 μm in diameter. To account for this size difference the area scaling factors (ASFs), and gains needed to be adjusted to make sure the fluorescence intensity was accurate and on scale. To maintain the accuracy of the fluorescence intensity, the height (H) and area (A) intensities needed to be equivalent at the peak channel of each cell. For example, the peak channel of mRuby3 is YG1, therefore the YG area scaling factor was adjusted until the fluorescent intensities of YG1-A and YG1-H were roughly the same. We repeated this for each of the eighteen pR-fp probes, adjusting the area scaling factors relative to the peak channel as needed. Since different fluorescent proteins with varying intensity levels were transfected at different DNA amounts, ranging from 50 ng to 300 ng per well in a 12-well plate (Table S10), the gains were adjusted to ensure that the fluorescence remained on scale. This adjustment was made until the brightest sample did not cause overflow in the peak channel. All the final setting parameters for pR-fp references are listed in Table S11. The normalized median fluorescence intensity (MFI) of the manually gated positive population of each pR-fp sample across 48 channels (Table S12) was used to profile the spectrum of that fp probe (Fig. S2). We verified that so long as the area scaling factors and gains remained constant, the fp spectra were constant.

### Nanopore Sequencing

#### pR-fp pool for native-barcoded nanopore sequencing

Approximately 4.5 picomoles of each pR-fp plasmid (equivalent to 1.05μg of DNA per 50μL reaction, across 10 reactions) were first treated with NheI-HF (*New England Biolabs*, Cat# R3131L) overnight, followed by an additional overnight digestion with MfeI-HF (*New England Biolabs*, Cat# R3589L) to achieve maximum digestion efficiency. After heat inactivation at 80°C for 20 minutes, all ten reactions of double-digested pR-fp fragments were pooled into a 2mL-DNA LoBind Eppendorf tube and supplemented with 1/10 v/v of sodium acetate (3M, pH5.2) (*Thermo Scientific*, Cat# R1181), 0.05μg/μL of Glycogen (Thermo Scientific, Cat# R0561), and 2.5 volumes of absolute ethanol (*Fisher Scientific*, Cat# BP2818-4). All tubes were inverted gently until fully mixed and incubated at −20°C overnight. After two rounds of centrifugation at 16,000*g* for 15 minutes at 4°C, the DNA pellets were air-dried for 10 minutes at room temperature and then dissolved in nuclease-free water. The concentrated fragments (NheI-CMV-fp-MfeI) were subsequently isolated using the Monarch Gel DNA Extraction Kit (New England Biolabs, Cat# T1020L). The concentration of each purified probe was measured by a Qubit Flex fluorometer (*Invitrogen by Thermo Fisher Scientific*) using a Qubit 1X dsDNA HS assay kit (*Invitrogen*, Cat# Q33231).

Approximately 250fmol of each digested fragment was subjected to end-prep with a NEBNext Ultra II End repair/dA-tailing Module (*New England Biolabs*, Cat# E7546S). They were then purified with the AMPure XP Beads provided from the native barcoding kit 24 (V14) (*Oxford Nanopore*, Cat# SQK-NBD114.24) and quantified via Qubit. Each probe was ligated with native barcodes from the nanopore native barcoding kit using the blunt/TA ligase master mix (*New England Biolabs*, Cat# M0367S). The ligation products from individual fluorescent probes were pooled and underwent a second round of purification using the AMPure XP Beads, followed by another Qubit quantification. The pooled probes were further ligated with the adapters from the native barcoding kit using the NEB quick T4 ligase (*New England Biolabs*, Cat# E6056S). After a third round of purification and quantification, approximately 20fmol of barcoded fluorescent probe library was prepared and loaded into a MinION flow cell (*Oxford Nanopore*, Cat# R10.4.1), which was then inserted into a MinION MK1C device (*Oxford Nanopore*) for nanopore sequencing.

#### Sequenced MuSIC Barcodes pool for native-barcoded nanopore sequencing

Twenty pMuSICs verified by sequencing (*Plasmidsaurus*) were selected for native-barcoded nanopore sequencing as shown in Table S13. Approximately 1.9 picomoles of each pMuSIC (equivalent to 1μg of DNA per 50μL reaction, across 3 reactions) were double digested with NheI-HF (*New England Biolabs*, Cat# R3131L) and MfeI-HF (*New England Biolabs*, Cat# R3589L) overnight. Each pMuSIC was end-prepped and ligated with a native barcode provided from the native barcoding kit 24 (V14) (*Oxford Nanopore*, Cat# SQK-NBD114.24) and mixed as a MuSIC barcodes pool prior to the adaptor ligation for the subsequent native-barcoded nanopore sequencing, as described above. Approximately 20fmol of barcoded sequenced pMuSIC library was prepared and loaded into a MinION flow cell (*Oxford Nanopore*, Cat# R10.4.1), which was then inserted into a MinION MK1C device (*Oxford Nanopore*) for nanopore sequencing.

#### MuSIC-barcode pool for nanopore sequencing

The MuSIC barcodes pool prepared by maxiprep was double-digested as 1μg of DNA per 50μL reaction, across 10 reactions, with NheI-HF (*New England Biolabs*, Cat# R3131L) and MfeI-HF (*New England Biolabs*, Cat# R3589L) overnight. The ligated products were heat inactivated at 80°C for 10 minutes and concentrated by ethanol precipitation for electrophoresis. DNA fragments (NheI-CMV-fp-tPT2A-fp-MfeI) containing MuSIC barcodes were isolated by the Monarch Gel DNA Extraction Kit (New England Biolabs, Cat# T1020L) and subject to end-prep, adapter ligation, multiple DNA cleanups and quantifications as described above to prepare the library for nanopore sequencing using a ligation sequencing kit V14 (*Oxford Nanopore*, Cat# SQK-LSK114). Approximately 20fmol of the pooled MuSIC barcode library was prepared and loaded into a MinION flow cell (*Oxford Nanopore*, Cat# R10.4.1), which was then inserted into a MinION MK1C device (*Oxford Nanopore*) for nanopore sequencing.

### Computational

#### Availability

The code and data used in this study are available upon request, and the code is publicly available on github (github.com/birtwistlelab/MuSIC-Fluorescent-Protein-Barcodes--Nanopore_Sequencing_Analysis and MuSIC-Fluorescent-Protein-Barcodes--Spectral_Unmixing).

#### Nanopore Sequencing Analysis

##### pR-fp pool for barcoded nanopore sequencing

Each sample probe, prepared with a nanopore native barcode, was considered as an *Actual* probe. Sequences in each barcode folder, derived from the Nanopore sequencing pass package, represented positive reads filtered according to these native barcodes. The universal coding workflow for nanopore sequencing is illustrated in Fig. S7A. Data were decompressed and filtered to retain only those within the expected length range of 1.3 to 1.6 kb, by selecting sequences between 1.2 and 1.8 kb. The filtered DNA sequences were reoriented to match the 5’->3’ direction of the CMV common cassette, using the *Align.PairwiseAligner* function in *global* alignment mode. Those with over 80% alignment to the CMV sequence were selected to minimize global scoring errors. These selected reads were then aligned and scored against the corresponding control sequences from a library containing 18 probe references (NheI-CMV-fp-MfeI), using the same alignment function.

For each sample probe, a score matrix of dimensions N x 18 was generated, where N represented the number of selected reads and 18 corresponded to the number of fp references. For each read, the highest score among the 18 references was assigned a value of 1, marking the most probable match, while the other probes for the same read were assigned a score of 0. This produced a binary scoring matrix of 1s and 0s. For each sample probe, scores were summed across the reads for each of the 18 references, resulting in a 1 × 18 vector representing the distribution of *sample reads*. The reference index with the highest scores was considered as the *inferred* probe index of this sample, while the reference fp with the highest score was identified as the *inferred* probe.

The positive percentage for each probe could be calculated by dividing the count of matches for each probe by the total number of barcoded reads, further generating the probe classification matrix (Table S14). The diagonal values of the 18 × 18 matrix were considered as the true positive rates (TPR) for each probe and used to construct an 18-*inferred* fp x 18-*actual* fp TPR heatmap (Fig. S7B), suggesting how well each fp could be identified by nanopore sequencing.

##### MuSIC barcodes pool for native-barcoded nanopore sequencing

Each sample barcode, labeled with a nanopore native barcode, was considered as an *actual* barcode. Sequences in each barcode folder from nanopore sequencing results were decompressed and filtered to retain only those within the expected length range of 2.1 to 2.5 kb, by selecting sequences between 2.0 and 2.8 kb. Following a similar procedure as described above, sequences were aligned to the CMV common cassette (5’->3’) to correct their direction. Only sequences with over 80% alignment were selected to minimize global scoring errors. These selected reads were then aligned and scored against a control library containing 324 references of MuSIC barcodes (NheI-CMV-fp-tPT2A-fp-MfeI), as the combination of 18 fp probes x 18 fp probes could generate 324 possible fp combos (considering order is necessary for sequence-level analysis).

For each sample barcode, a score matrix of dimensions N x 324 was generated, where N represented the number of selected reads and 324 corresponded to the number of MuSIC barcode references. For each read, the highest score among the 324 references was assigned a value of 1, marking the most probable match, while the remaining barcodes for that read were assigned a score of 0. This also produced a binary scoring matrix of 1s and 0s. For each sample barcode, scores were summed across the reads for each of the 324 references, resulting in a 1 × 324 vector representing the distribution of sample reads. The reference index with the highest scores was considered as the *inferred* barcode index of this sample, while the reference fp combo with the highest score was identified as the *inferred* barcode.

The positive percentage for each barcode was calculated by dividing the count of matches for each barcode by the total number of the native barcode-labeled reads, resulting in a 20-pMuSIC x 324-barcode matrix. This matrix was subsequently used to generate a 20 actual-pMuSIC x 20 inferred-barcode classification matrix (Table S15). The diagonal values of this matrix, representing TPR for each barcode, were used to construct a 20 × 20 heatmap (Fig. S7C), indicating the effectiveness of identifying MuSIC barcodes in a mixture based on nanopore sequencing.

##### MuSIC-barcode pool for nanopore sequencing

Data from the nanopore sequencing pass package were decompressed and filtered to include only those within the 2.1 to 2.7 kb length range, by selecting reads between 2.0 and 3.0 kb. Each read was aligned to the CMV common cassette and reoriented to the 5’->3’ direction. Only those with over 80% alignment to the CMV sequence were selected to minimize global scoring errors. The selected reads were then aligned to a control library containing 324 MuSIC barcodes (NheI-CMV-fp-tPT2A-fp-MfeI). Similarly, a score matrix of dimensions N x 324 was generated, where N represented the number of filtered reads. The highest score among the 324 barcodes for each read was assigned a value of 1, indicating the most probable MuSIC barcode, while all other scores were assigned 0. This resulted in a binary scoring matrix of 1s and 0s. The scores were summed along each column to generate a vector dataset with dimensions 1 × 324, which was then used for calculating the relative abundance of each barcode by dividing the number of the total reads. The data was reshaped into an 18×18 matrix (Table S16) to generate an 18-fp x18-fp heatmap for better visualization of the fractions of MuSIC barcodes. A total of approximately 323 barcodes were identified in the unmixed 18×18 matrix, excluding pMuSIC-miRFP670-CyOFP1 (Table S16). However, sequencing by Plasmidsaurus (Table S17) confirmed that pMuSIC-S94 was indeed pMuSIC-miRFP670-CyOFP1, indicating that all pMuSIC barcodes were present in pMuSIC-barcode pool.

#### Spectral unmixing analysis

Spectral data FCS files, containing fluorescence intensities of each cell across 48 channels from the Cytek Aurora, were converted to CSV files with scale values using FlowJo v10.10.0 software (*BD Life Science*). The unmixing workflow of spectral data analysis is illustrated in Fig. S8.

##### Spectral unmixing for single fp probes

A subset of the positive cell population with moderately high fluorescence intensity (FI) of each probe was selected to determine the reference spectra, calculated from the median fluorescence intensity for each channel.

Spectral data for the entire population of each pR-fp group, referred as the pR-fp singlets, were analyzed to create a histogram of fluorescence intensity (FI) versus frequency. Based on the cell population of the negative control, the whole positive population of each fp probe was separated from the negative population. The spectral data of this positive population were unmixed with the MFI reference of each probe using non-negative least squares (NNLS). The unmixed result for each pR-fp was an N by 18 matrix, where N is the number of cells, and 18 is the number of fluorescent proteins. The reference with the highest value is the most likely fp probe in the sample. Frequency versus intensity was further analyzed, generating FPR, TPR and ROC analyses for each fp probe. To balance a high TPR and a low FPR, the optimal threshold for each fp probe was determined by identifying the point on the ROC curve closest to the ideal one (FPR=0, TPR=1). These thresholds for 18 fp probes were applied to distinguish false positive cells from true positive cells under four different conditions: original, intensity cutoff, remove mTFP1, as well as intensity cutoff and remove mTFP1.

Under the original condition, the percentage of false positive cells within the positive population of each fp probe was used to generate the 18-fp x 18-fp FPR heatmap (Fig.3C), while the numbers of false positive cells were used to illustrate how low FI may contribute to unmixing errors (Fig.3D). The FPRs under four different conditions were summarized in Fig.3E.

##### Spectral unmixing for MuSIC barcodes

The positive population of each sequenced MuSIC barcode (*actual*) was manually gated based on the peak channel of the singlet spectrum. Spectral data were converted to CSV file, as described above. Eighteen fp probes could generate a control library with 153 unique combinations, regardless of order (i.e., combinations rather than permutations), while seventeen fp probes could generate a library with 136 unique combinations.

Each cell in each sample was unmixed to identify the most likely fp combination (*inferred*) in the control library, using the NNLS method with the 18 references under four different conditions: original, intensity cutoff, remove mTFP1, as well as intensity cutoff and remove mTFP1. The vector output of unmixing for each cell, with a shape of (1, 18) were generated, which were then divided by the 18 thresholds determined by identifying the point on the ROC curve of fluorescent probes as described above. This process generated a normalized score vector (1 × 18), optimizing classification accuracy and enabling direct comparison between different fluorescent probes. The fp references with the top two highest scores were considered the most likely fluorescent probes constituting the fp combination for that MuSIC barcode. Thus, an inferred barcode of each cell could be identified.

If the inferred barcode of a cell matches the actual barcode of that sample determined by sequencing (Table S17), it was considered a true positive; otherwise, it would be a false positive. For each singly transfected pMuSIC sample, TPR and FPR were calculated. An average TPR was computed for the replicates of each pMuSIC sample. When two different MuSIC barcodes with inverted orientations of fluorescent proteins correspond to the same fluorescent protein combination, their average TPR was used. Therefore, a triangle heatmap of TPR illustrating all singly transfected pMuSICs was created under each condition.

## Supplementary Material

Supplement 1

Supplement 2
