# Supplementary material for: Genetically-Encoded Fluorescence Barcodes Allow for Single-Cell Analysis via Spectral Flow Cytometry
Source: bioRxiv. 2025 Mar 28:2024.10.23.619855. Originally published 2024 Oct 24. Preprint. [Version 2] doi: 10.1101/2024.10.23.619855 (PMC11526929; doi:10.1101/2024.10.23.619855)
Supplement: 1 [file NIHPP2024.10.23.619855V2-supplement-1.pdf]

bioRxiv preprint doi: https://doi.org/10.1101/2024.10.23.619855; this version posted March 28, 2025. The copyright holder for this preprint (which was not certified by peer review) is the author/funder, who has granted bioRxiv a license to display the preprint in perpetuity. It is made available under aCC-BY 4.0 International license.

**Supporting Information for:**

# Genetically-Encoded Fluorescence Barcodes Allow for Single-Cell Analysis via Spectral Flow Cytometry

Xiaoming Lu*[,1], Daniel J. Pritko*[,1], Megan E. Abravanel[1], Jonah R. Huggins[1], Oluwaferanmi Ogunleye[1], Tirthankar Biswas[1], Katia C. Ashy[1], Semaj K. Woods[1], Mariclaire W.T. Livingston[1], Mark A. Blenner[2,#], Marc R. Birtwistle[1,#]

*Equal contribution

[#]correspondence: mbirtwi@clemson.edu; blenner@udel.edu

[1]Department of Chemical and Biomolecular Engineering, Clemson University

[2]Department of Chemical and Biomolecular Engineering, University of Delaware

bioRxiv preprint doi: https://doi.org/10.1101/2024.10.23.619855; this version posted March 28, 2025. The copyright holder for this preprint (which was not certified by peer review) is the author/funder, who has granted bioRxiv a license to display the preprint in perpetuity. It is made available under aCC-BY 4.0 International license.

**Figure S1, Lu et al**

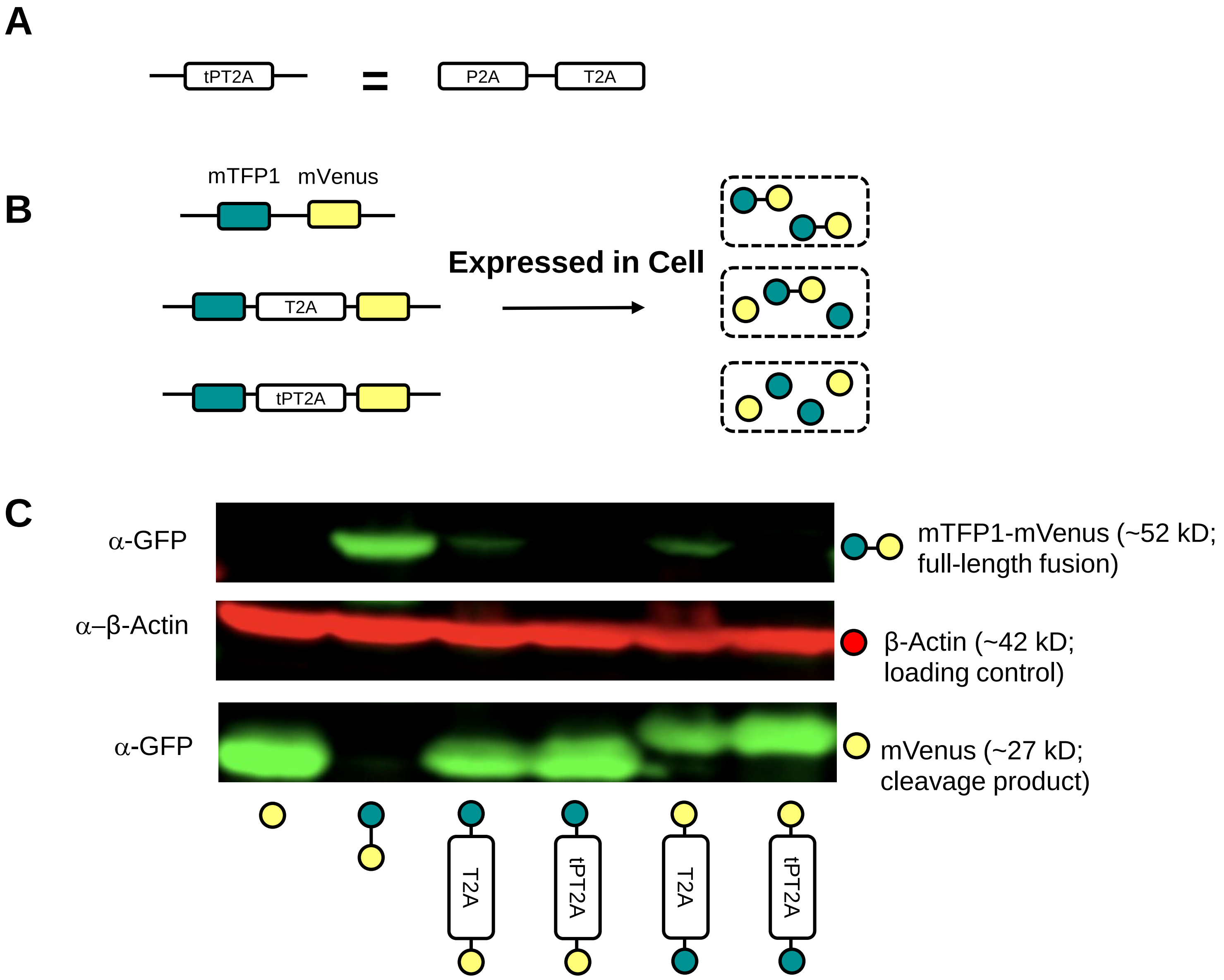


**Figure S1. Performance of the Tandem P2A-T2A for Cleaving Barcode Elements. (A)** tPT2A is a tandem sequence of the P2A and T2A sequences. **(B)** T2A and tPT2A sequences can be used to prevent the fusion of fluorescent proteins expressed by the same plasmid within a cell. **(C)** Western blot comparing the effectiveness of T2A and tPT2A in preventing the fusion of fluorescent proteins in a barcode. HEK293T cells were transfected with indicated plasmids containing variations of mTFP1 and mVenus, then lysates harvested and subjected to western blotting. The anti-GFP antibody recognizes mVenus. Residual fusion protein is not detectable with tPT2A.

bioRxiv preprint doi: https://doi.org/10.1101/2024.10.23.619855; this version posted March 28, 2025. The copyright holder for this preprint (which was not certified by peer review) is the author/funder, who has granted bioRxiv a license to display the preprint in perpetuity. It is made available under aCC-BY 4.0 International license.

**Figure S2 Lu et al**

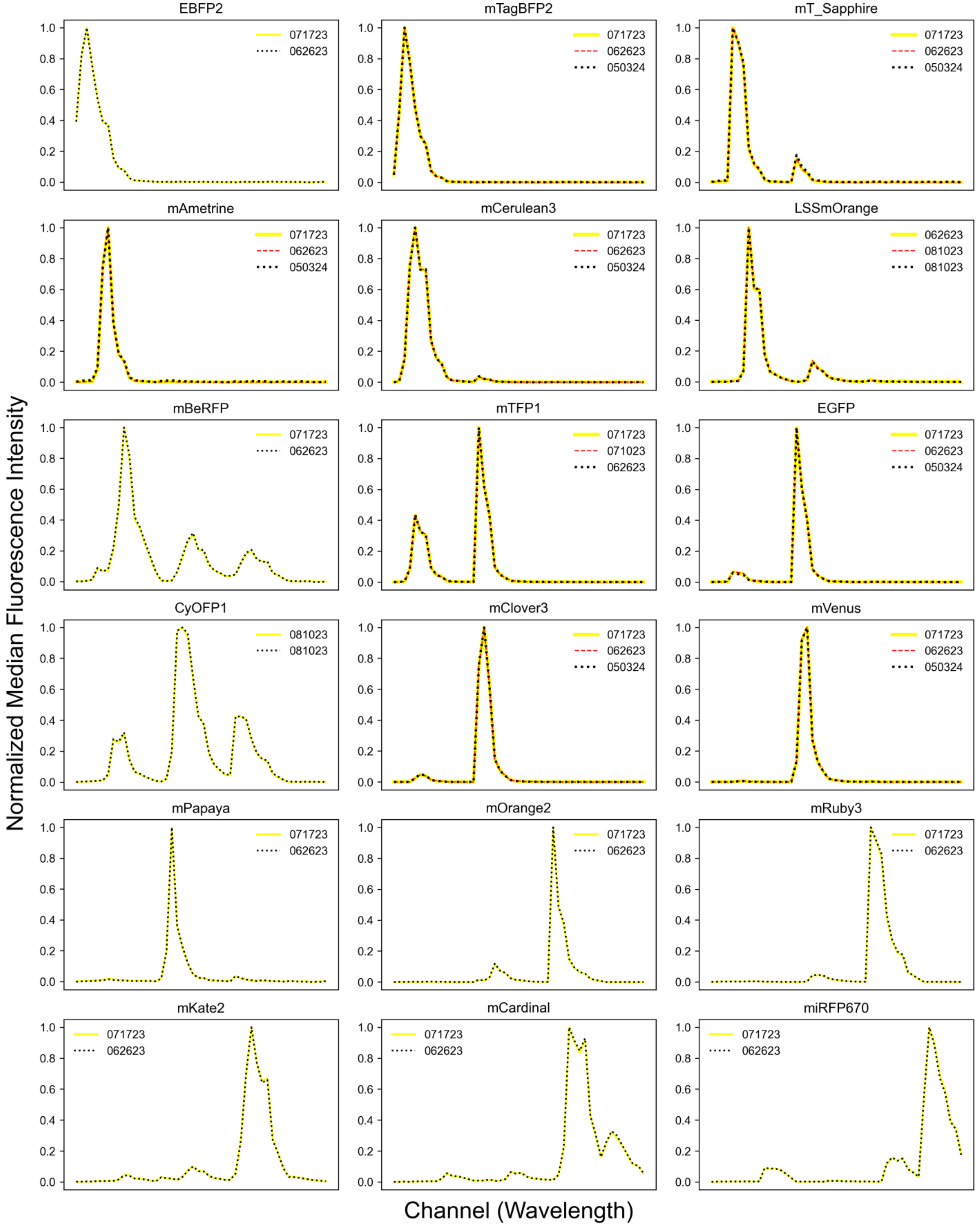


**Figure S2. Fluorescent Protein Emission Spectra.** HEK293T cells were transfected with plasmids encoding each of the 18 fluorescent proteins(FP), and the normalized median emission intensity spectra were measured using spectral flow cytometry. At least two replicates were performed for each pR-FP probe. In each subplot, different replicates are represented by different colors, with their respective dates used to identify them in the legend.

bioRxiv preprint doi: https://doi.org/10.1101/2024.10.23.619855; this version posted March 28, 2025. The copyright holder for this preprint (which was not certified by peer review) is the author/funder, who has granted bioRxiv a license to display the preprint in perpetuity. It is made available under aCC-BY 4.0 International license.

**Figure S3 Lu et al**

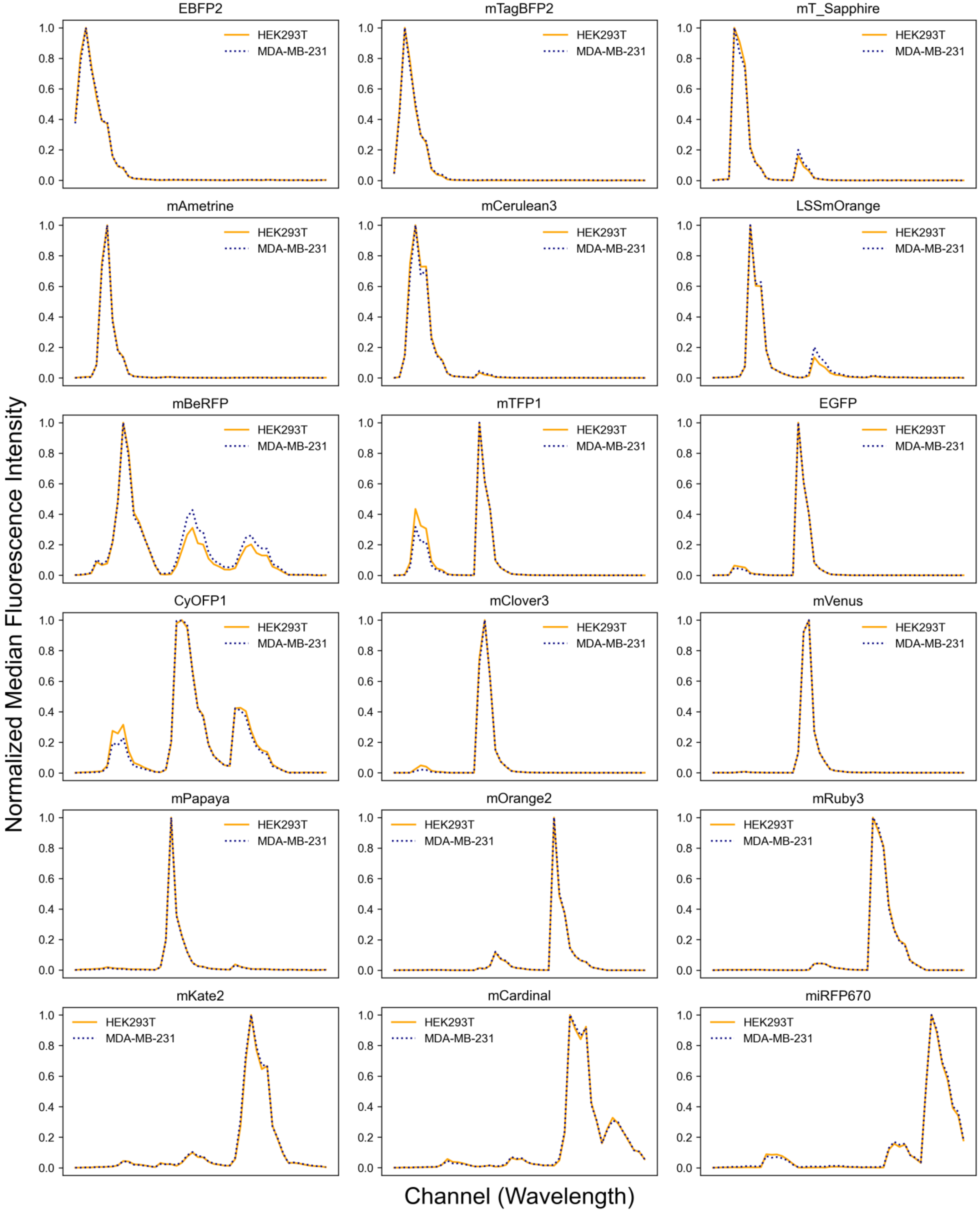


**Figure S3. Fluorescent Protein Emission Spectra in HEK293T and MDA-MB-231 Cells**. Both cell lines were transfected with plasmids encoding each of the 18 fluorescent proteins, and their normalized median fluorescence intensity spectra were measured using spectral flow cytometry. Each spectra is the average from at least two replicates.

bioRxiv preprint doi: https://doi.org/10.1101/2024.10.23.619855; this version posted March 28, 2025. The copyright holder for this preprint (which was not certified by peer review) is the author/funder, who has granted bioRxiv a license to display the preprint in perpetuity. It is made available under aCC-BY 4.0 International license.

# Figure S4 , Lu et al

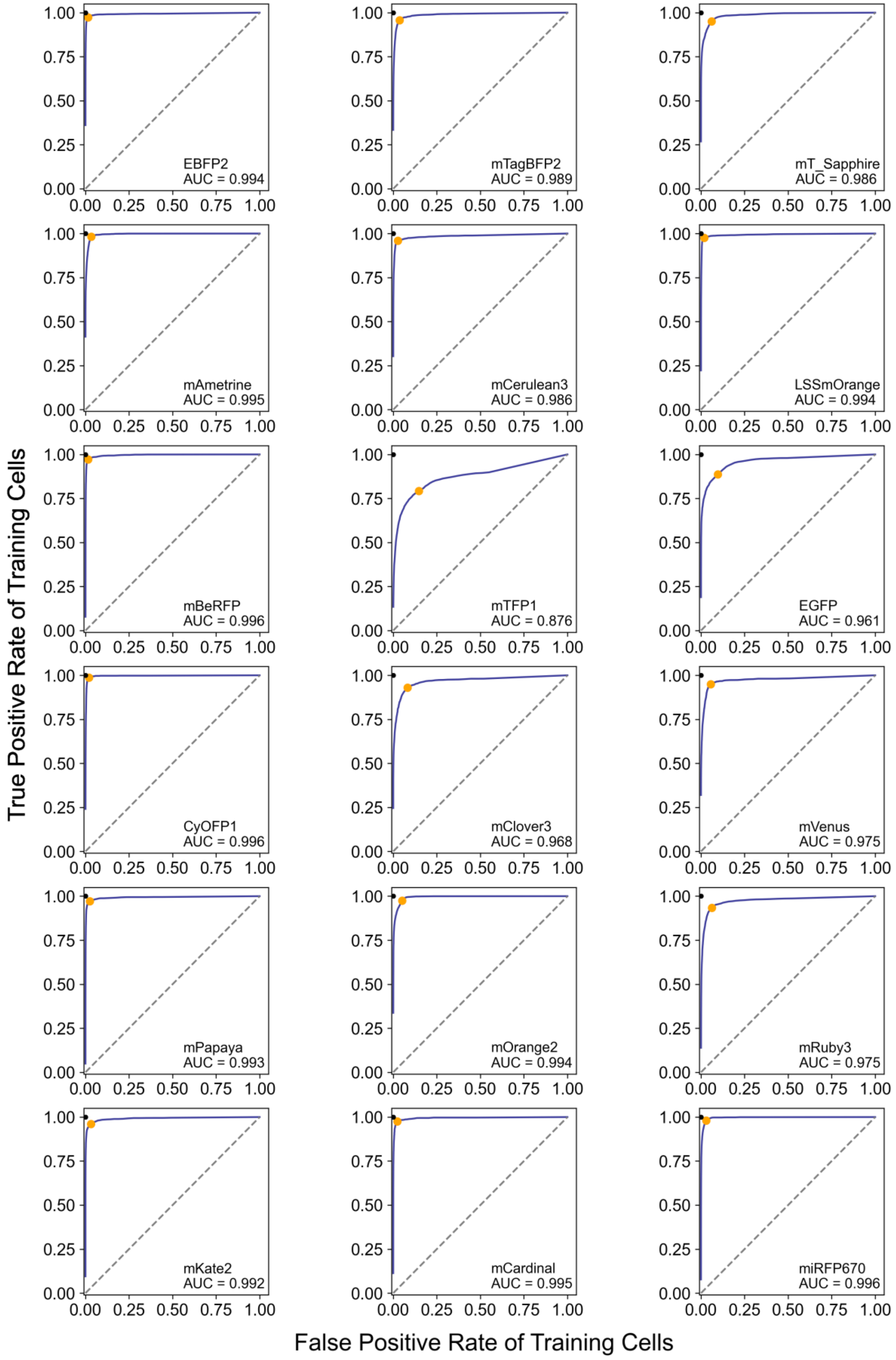


**Figure S4. Threshold Estimation for Each Fluorescence Protein from Training Data.** For varying thresholds, the training data underwent unmixing, thresholding, and classification to compute the true positive and false positive rates, enabling the construction of a receiver operating characteristic (ROC) curve for each fluorescent protein. The area under the curve (AUC) is displayed in each inset, along with the chosen threshold point (orange) which balances both false positive rate and true positive rate.

bioRxiv preprint doi: https://doi.org/10.1101/2024.10.23.619855; this version posted March 28, 2025. The copyright holder for this preprint (which was not certified by peer review) is the author/funder, who has granted bioRxiv a license to display the preprint in perpetuity. It is made available under aCC-BY 4.0 International license.

# Figure S5. Lu et al

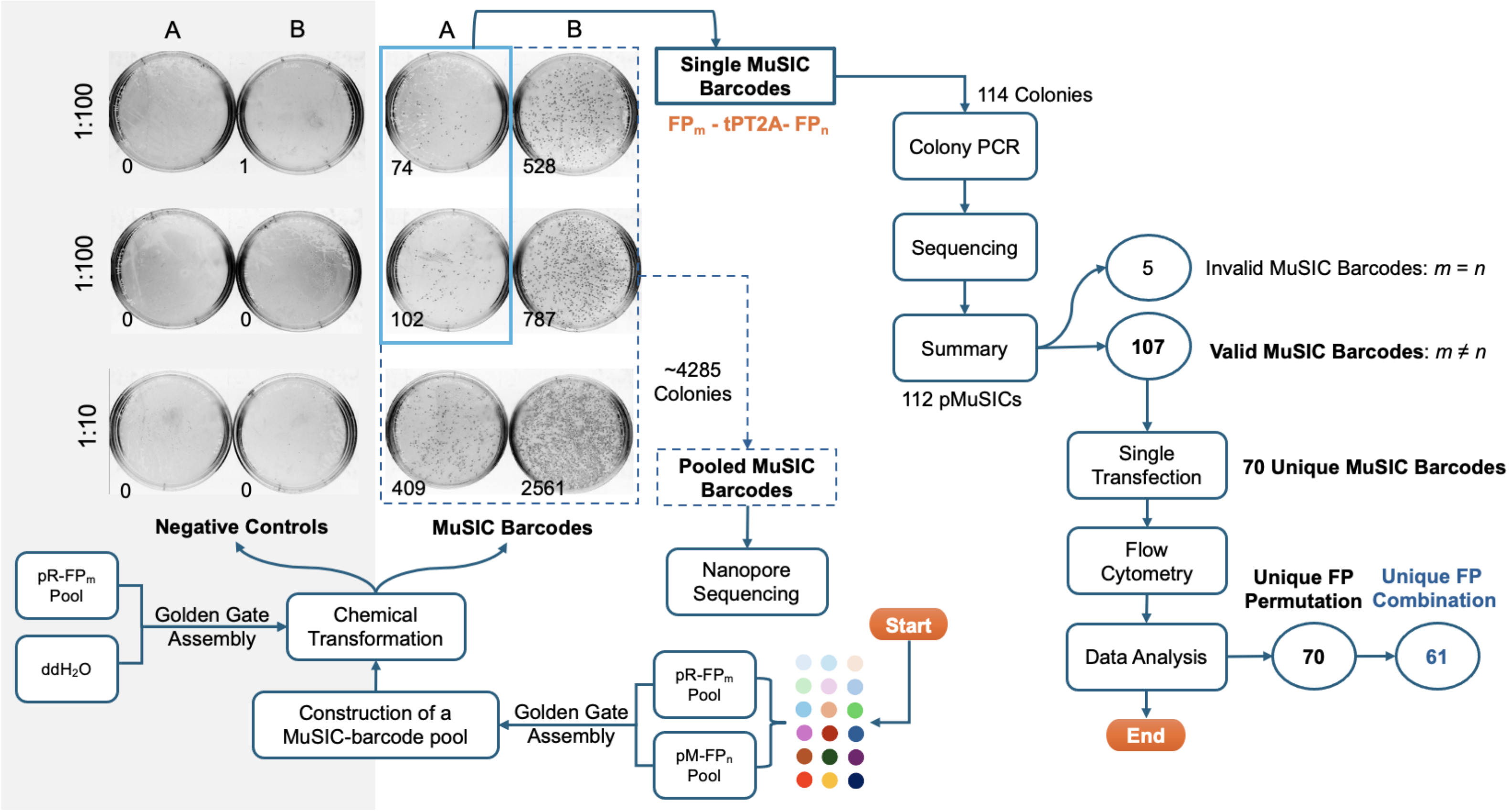


**Figure S5. Workflow for Constructing and Analyzing Barcodes**. This flowchart outlines the process of constructing the barcode pool through chemical transformation, generating single barcodes for flow cytometry analysis and pooled MuSIC barcodes for nanopore sequencing. To better isolate single colonies, we performed duplicate transformations using a 1:100 dilution of the GoldenGate assembly product. To maximize the colonies without compromising the transformation efficiency, a 1:10 dilution was also performed. We used $ddH_2O$ in place of the insert pool as the negative control. The number in the bottom left corner of each plate represents the colony count. More than 4000 colonies from the MuSIC-barcode plates, highlighted by the navy dotted square, were scraped and pooled to create the MuSIC barcode library for nanopore sequencing. In the sky blue solid square, 114 colonies were selected and screened by colony PCR and then sequencing (*PlasmidSaurus*). Among 112 positive pMuSICs, 107 contained different FPs and thus were considered valid. Of these, 70 were unique barcodes based on permutation, and 61 were unique combinations.

bioRxiv preprint doi: https://doi.org/10.1101/2024.10.23.619855; this version posted March 28, 2025. The copyright holder for this preprint (which was not certified by peer review) is the author/funder, who has granted bioRxiv a license to display the preprint in perpetuity. It is made available under aCC-BY 4.0 International license.

Figure S6, Lu et al

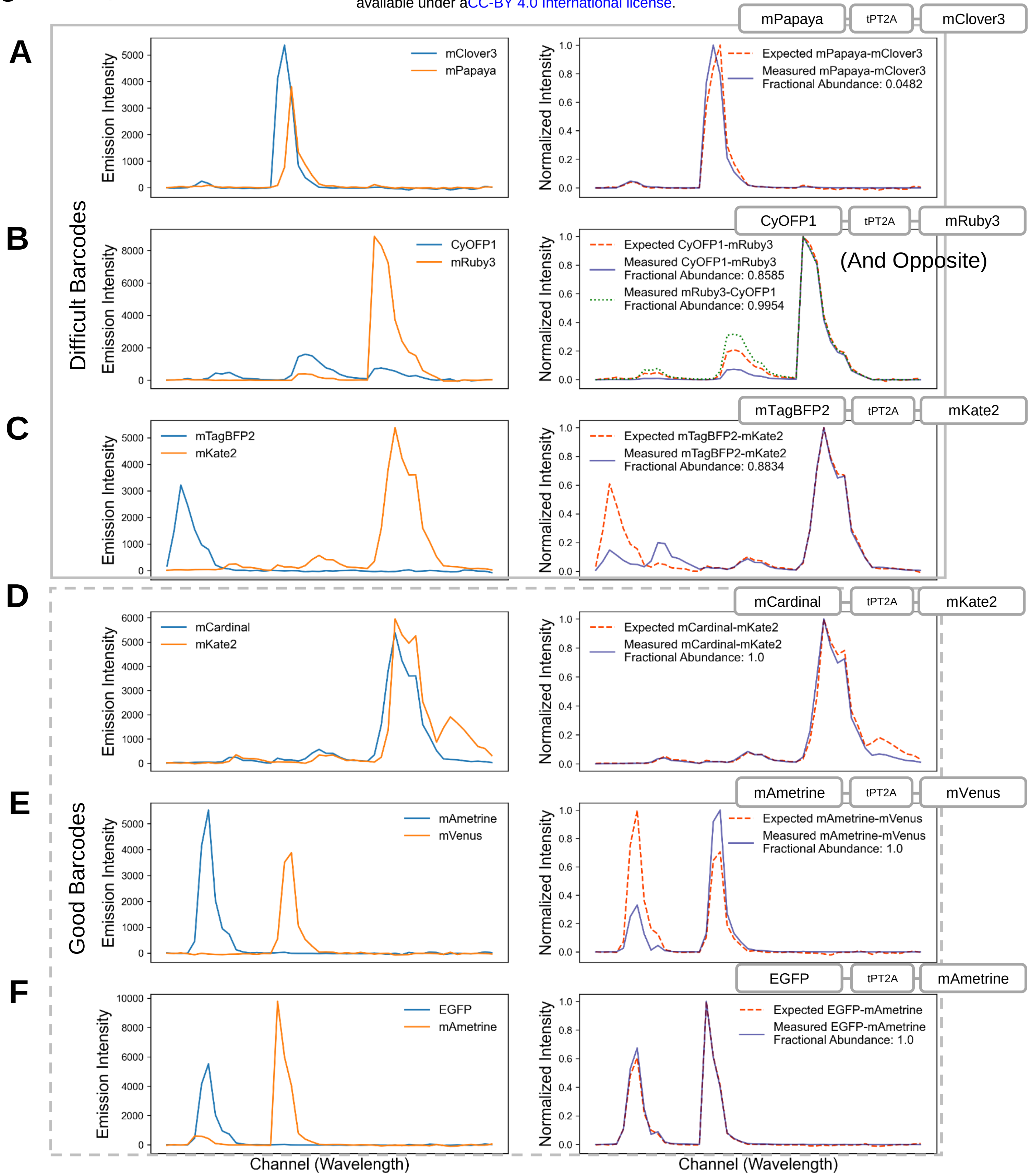


**Figure S6. Spectra of Difficult Barcodes (A-C) and Good Barcodes (D-F).** (**Left**) Raw intensity median spectra of individual fluorescent proteins (FPs). The raw intensity provides insight into brightness differences (if applicable). (**Right**) Normalized barcode "expected" spectra based on equal expression of the FPs on the left, and experimentally measured spectra. Fractional abundance is the proportion of correctly classified cells. Normalized intensity helps to compare between expected and experimentally measured—brightness differences are built-in. The order of each barcode in the plasmid is depicted at the top right. (**A**) mPapaya-mClover3. (**B**) CyOFP1-mRuby3, and the alternative permutation mRuby3-CyOFP1. (**C**) mTagBFP2-mKate2. (**D**) mCardinal-mKate2. (**E**) mAmetrine-mVenus. (**F**) EGFP-mAmetrine.

bioRxiv preprint doi: https://doi.org/10.1101/2024.10.23.619855; this version posted March 28, 2025. The copyright holder for this preprint (which was not certified by peer review) is the author/funder, who has granted bioRxiv a license to display the preprint in perpetuity. It is made available under aCC-BY 4.0 International license.

# Figure S7, Lu et al

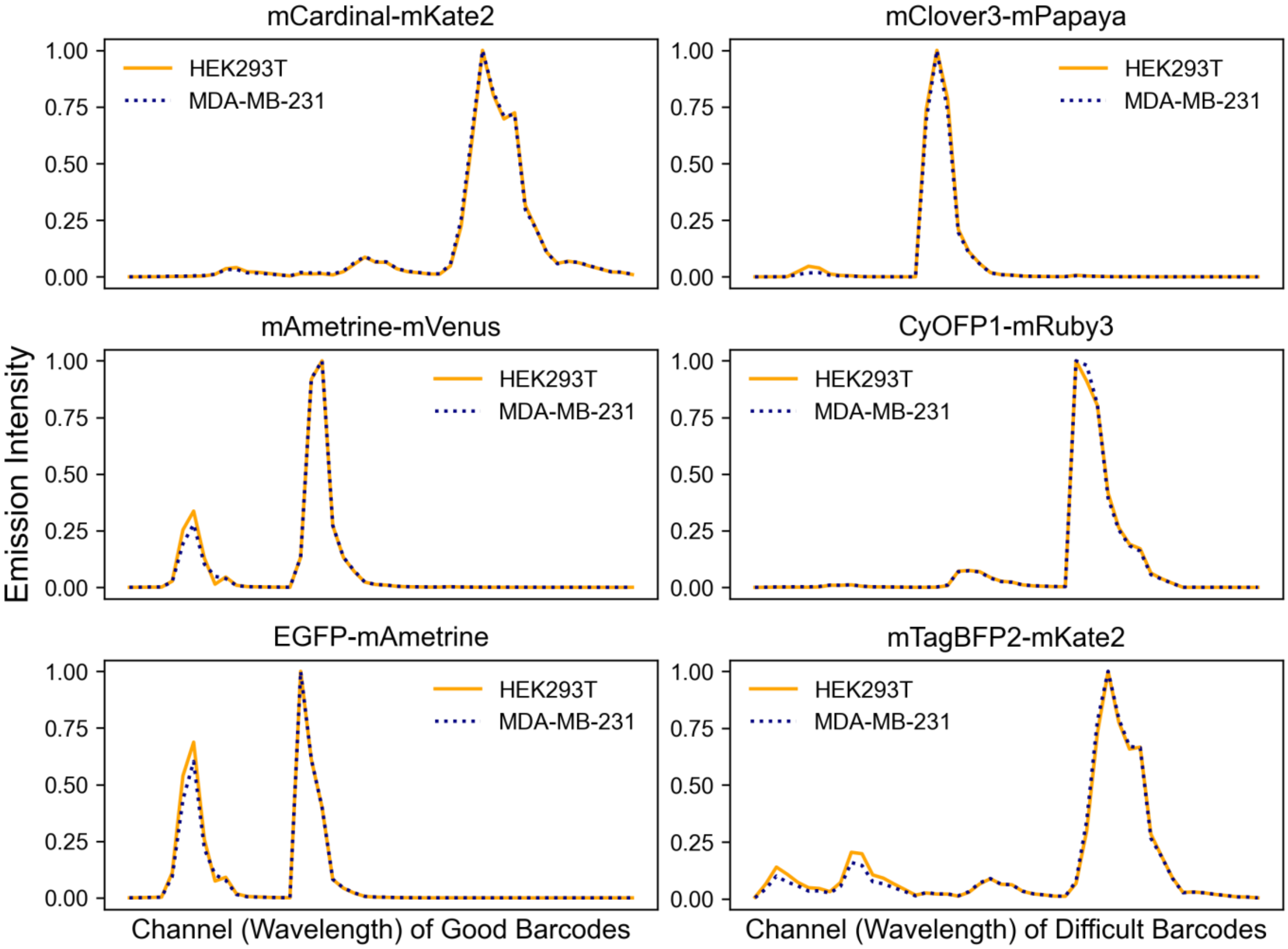


**Figure S7. Spectra of Good (Left) or Difficult (Right) Barcodes in Different Cell Lines.** Spectra were generated and analyzed as in Figure S6. Briefly, HEK293T or MDA-MB-231 cells were transfected with the barcodes as indicated then analyzed by spectral flow cytometry. Spectra are nearly identical in both cell lines.

bioRxiv preprint doi: https://doi.org/10.1101/2024.10.23.619855; this version posted March 28, 2025. The copyright holder for this preprint (which was not certified by peer review) is the author/funder, who has granted bioRxiv a license to display the preprint in perpetuity. It is made available under aCC-BY 4.0 International license.

# Figure S8, Lu et al

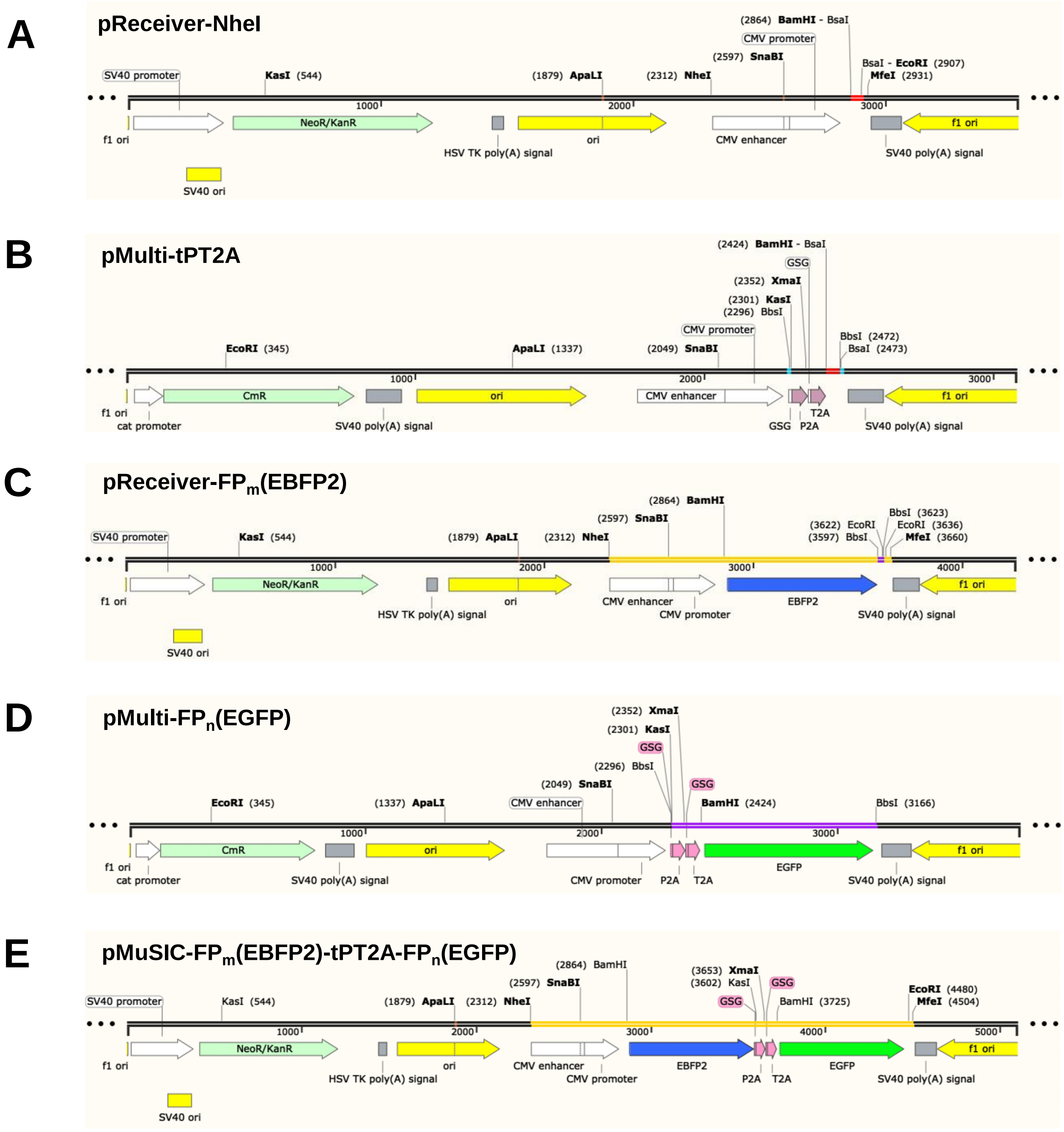


**Figure S8. Schematic Representations of Plasmid Backbones, FPs, and a MuSIC Barcode.** The schematic plasmid maps were generated using SnapGene. (**A**) An NheI site was inserted upstream of the CMV promoter in pReceiver to create pReceiver-NheI. A pair of BsaI sites (red) was used for insertion of an individual fluorescent protein (FP) as the first probe. (**B**) A pair of BbsI sites (light blue) was introduced upstream of T2A (in pMulti) or P2A-T2A (in pMulti-tPT2A, shown here) and downstream of the BsaI-spacer-BsaI FP insertion cassette (red). This design allowed the insertion of a second FP into pReceiver-FP to generate MuSIC barcodes, while the BsaI sites facilitated individual FP insertion. (**C**) An individual example $FP_m$ (EBFP2) was inserted into pReceiver-NheI via BsaI sites (as shown in **A**) to generate pReceiver-$FP_m$. A BbsI-TAA-BbsI cassette (purple) was included to terminate transcription for individual testing, and enable the loading of a second FP for MuSIC barcode generation. (**D**) An individual example $FP_n$ (EGFP) was inserted into pMulti-tPT2A via BsaI sites (as shown in **B**), generating pMulti-$FP_n$. The second FP, containing tPT2A and $FP_n$, is highlighted in purple. (**E**) The pMuSIC construct contains $FP_m$-tPT2A-$FP_n$, exemplified here as EBFP2-tPT2A-EGFP. NheI and MfeI sites in **C** and **E** were used to generate fragments (orange) for nanopore sequencing.

bioRxiv preprint doi: https://doi.org/10.1101/2024.10.23.619855; this version posted March 28, 2025. The copyright holder for this preprint (which was not certified by peer review) is the author/funder, who has granted bioRxiv a license to display the preprint in perpetuity. It is made available under aCC-BY 4.0 International license.

# Figure S9 Lu et al

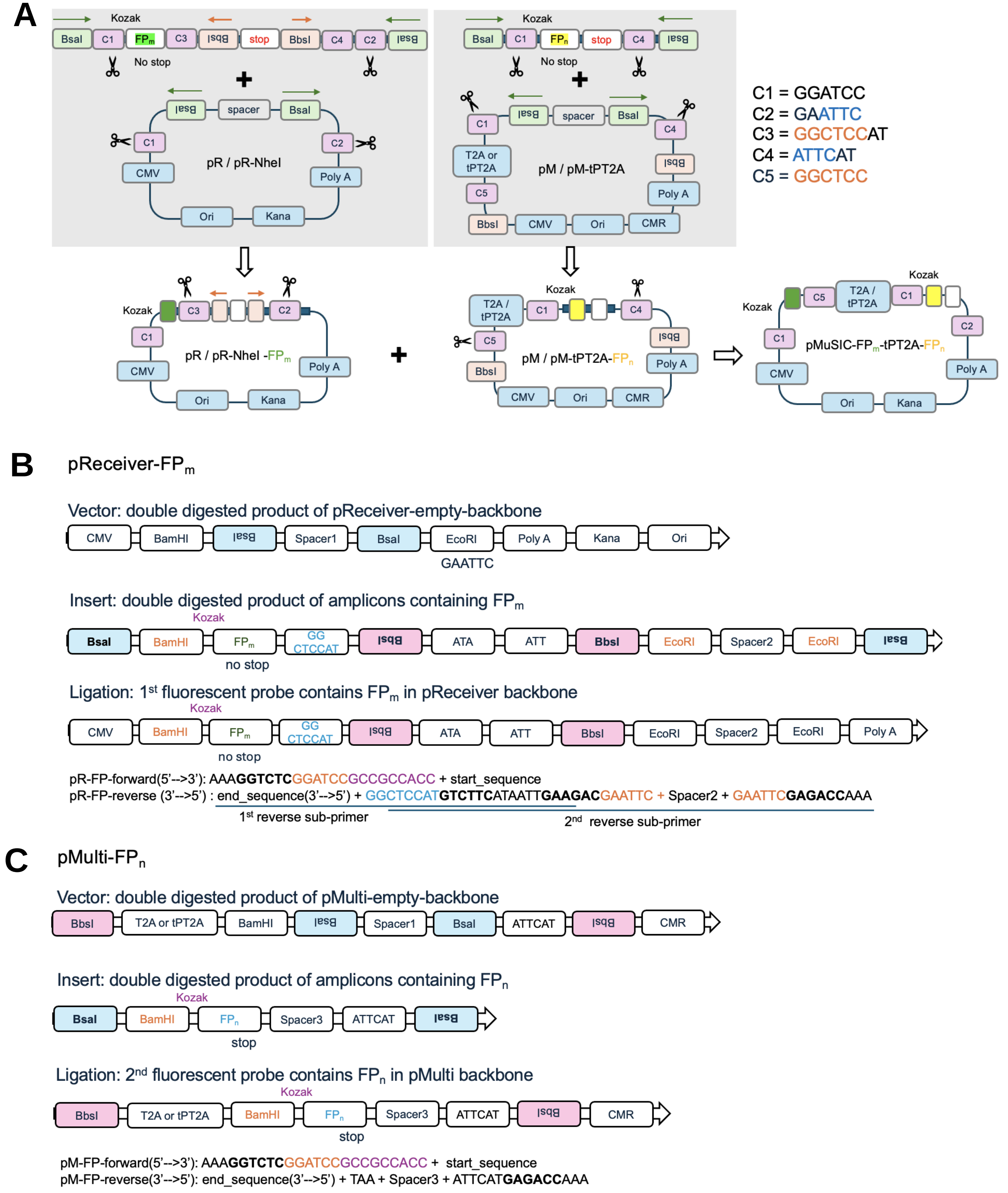


**Figure S9. pMuSIC Construction.** (**A**) Constructing pMuSIC by Goldengate. First, the BsaI-Spacer-BsaI cassettes in pR were removed by BbsI digestion, and the PCR products containing a fluorescent protein (FP, index *m*) and BbsI-TAA-BbsI cassette (stop), were inserted to create the vector plasmid pR-NheI-$FP_m$-BbsI-TAA-BbsI. Similarly, pM underwent BsaI digestion for the FP insertion (FP, index *n*), resulting in the insert plasmids pM-$FP_n$. Finally, both vector and insert plasmids were digested at BbsI sites to generate pMuSICs containing the MuSIC barcode ($FP_m$-2A-$FP_n$). (**B-C**) Primer designs to amplify the FP inserts for both the vector and insert plasmids, respectively. The sequences of all 18 FPs were used as templates (**Table S5**), with their start and end sequences listed in **Table S6**, along with the sequences of the spacers.

bioRxiv preprint doi: https://doi.org/10.1101/2024.10.23.619855; this version posted March 28, 2025. The copyright holder for this preprint (which was not certified by peer review) is the author/funder, who has granted bioRxiv a license to display the preprint in perpetuity. It is made available under aCC-BY 4.0 International license.



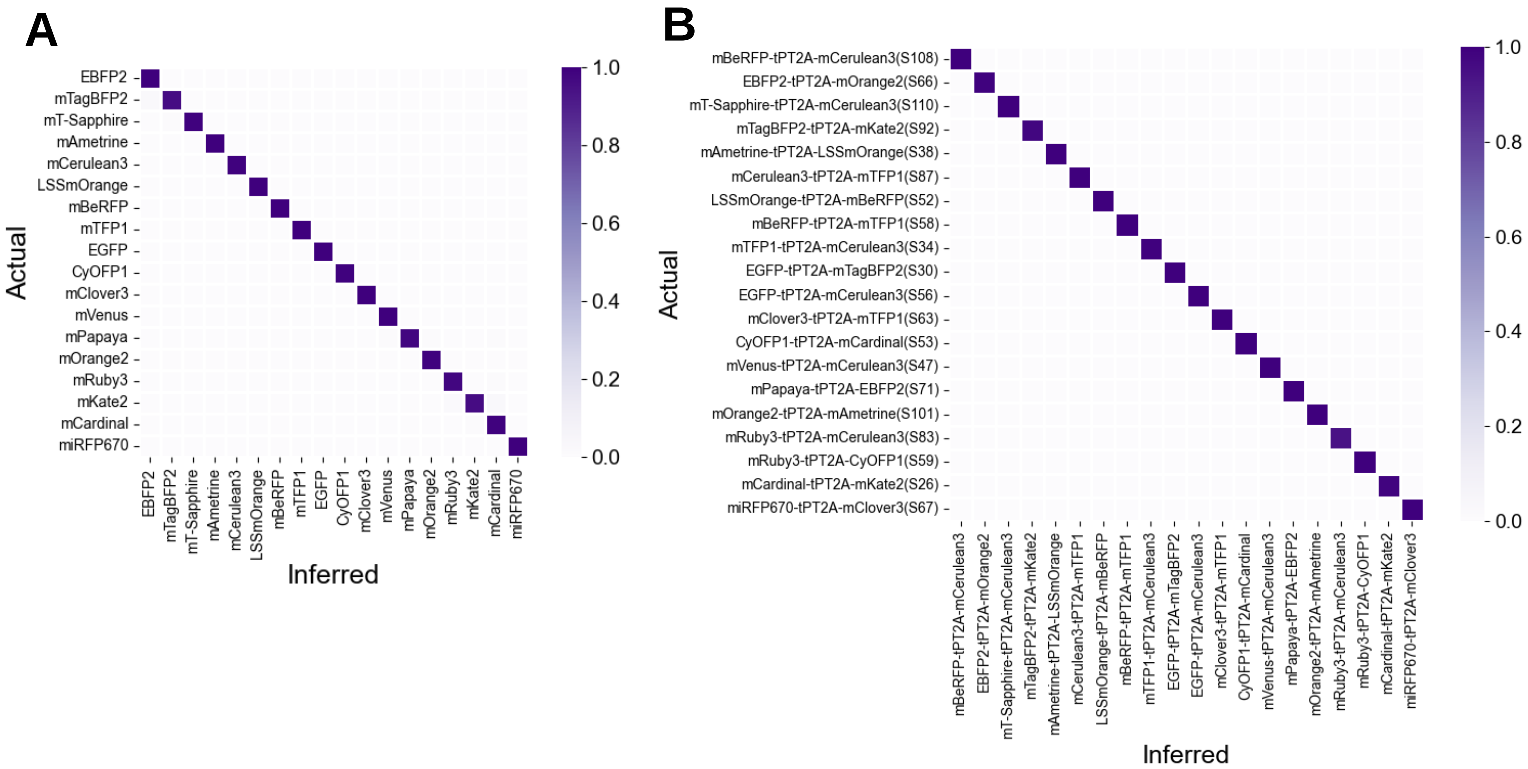


**Figure S10. Nanopore Sequencing Validation Results.** Classification of (**A**) 18 pR-FPs and (**B**) 20 barcodes Color bar denotes fraction inferred.
