# Supplementary material for: Genetically-Encoded Fluorescence Barcodes for Single-Cell Analysis
Source: bioRxiv. 2024 Oct 24:2024.10.23.619855. Preprint. [Version 1] doi: 10.1101/2024.10.23.619855 (PMC11526929; doi:10.1101/2024.10.23.619855)
Supplement: Supplementary file 1 [file NIHPP2024.10.23.619855v1-supplement-2.pdf]

bioRxiv preprint doi: https://doi.org/10.1101/2024.10.23.619855; this version posted October 24, 2024. The copyright holder for this preprint (which was not certified by peer review) is the author/funder, who has granted bioRxiv a license to display the preprint in perpetuity. It is made available under aCC-BY 4.0 International license.

Figure S1, Lu et al

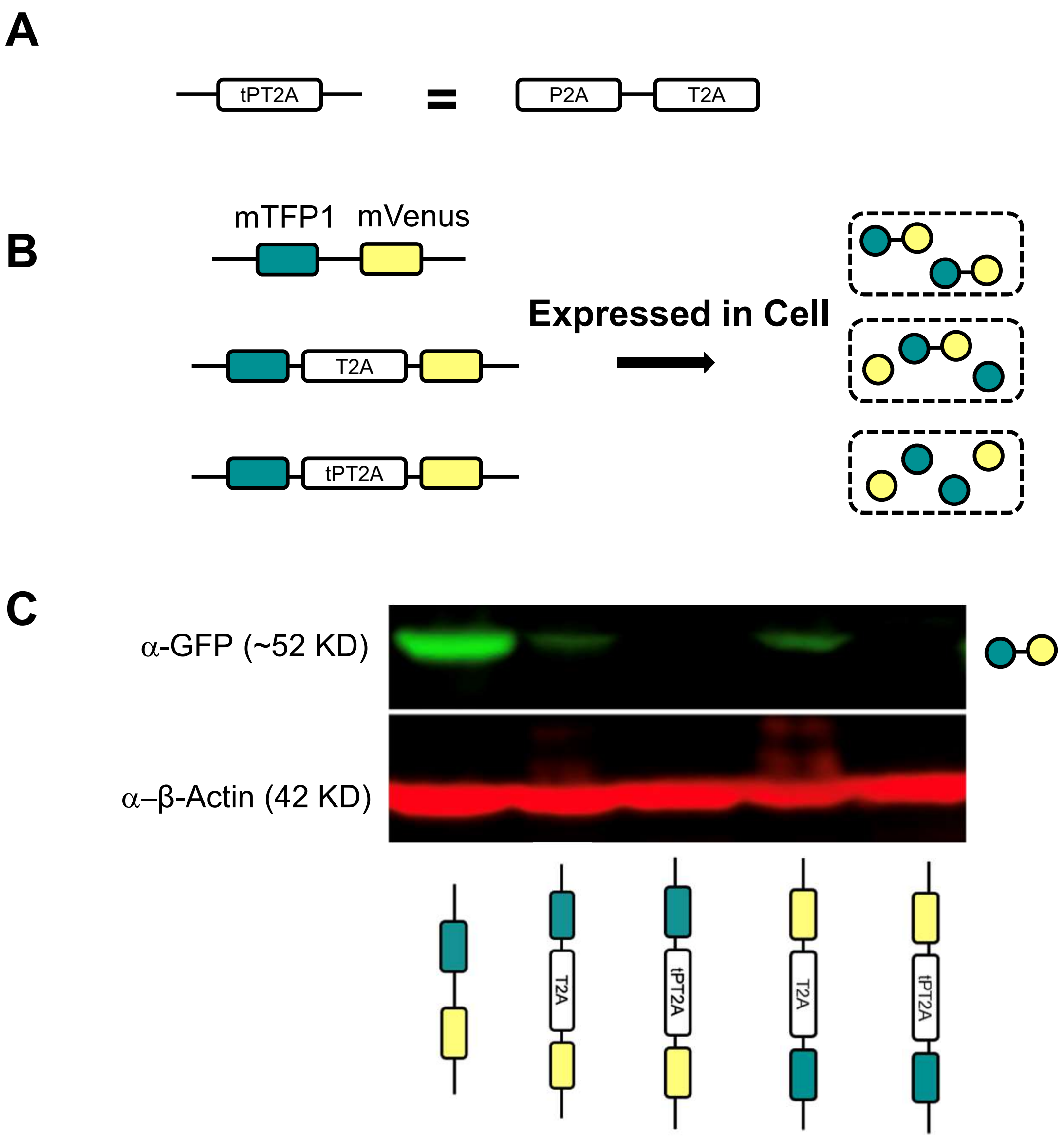

Figure S1. Performance of the Tandem P2A-T2A for Cleaving Barcode Elements. (A) tPT2A is a tandem sequence of the P2A and T2A sequences. **(B)** T2A and tPT2A sequences can be used to prevent the fusion of fluorescent proteins expressed by the same plasmid within a cell. **(C)** Western blot comparing the effectiveness of T2A and tPT2A in preventing the fusion of fluorescent proteins in a barcode. HEK293 cells were transfected with indicated plasmids containing variations of mTFP1 and mVenus, then lysates harvested and subjected to western blotting. The anti-GFP antibody recognizes mVenus. Residual fusion protein is not detectable with tPT2A.

bioRxiv preprint doi: https://doi.org/10.1101/2024.10.23.619855; this version posted October 24, 2024. The copyright holder for this preprint (which was not certified by peer review) is the author/funder, who has granted bioRxiv a license to display the preprint in perpetuity. It is made available under aCC-BY 4.0 International license.

Figure S2 Lu et al

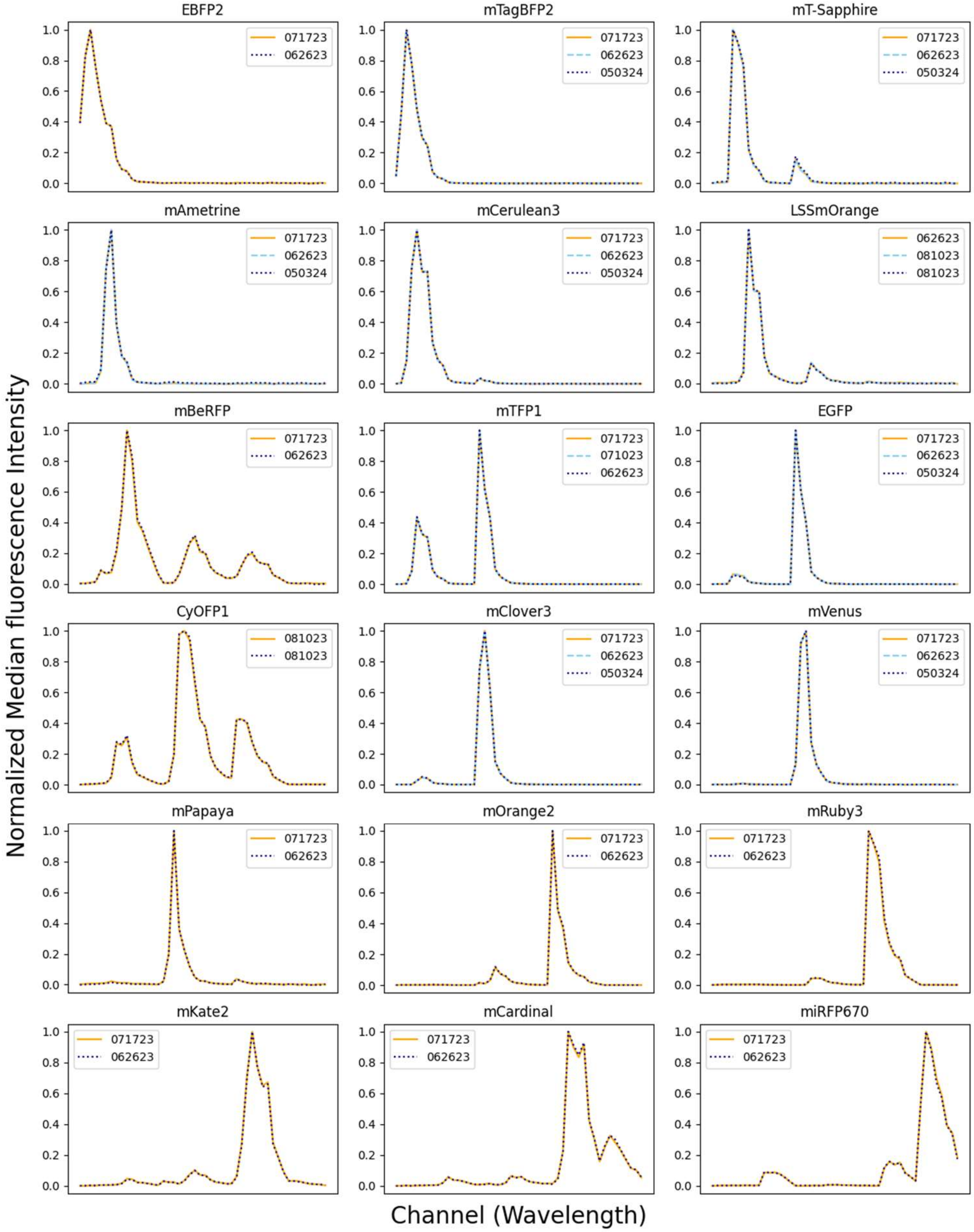

Figure S2. Fluorescent Protein Emission Spectra. HEK293 cells were transfected with plasmids encoding each of the 18 fluorescent proteins, and the normalized median emission intensity spectra were measured using spectral flow cytometry. At least two replicates were performed for each pR-fp probe. In each subplot, different replicates are represented by different colors, with their respective dates used to identify them in the legend.

bioRxiv preprint doi: https://doi.org/10.1101/2024.10.23.619855; this version posted October 24, 2024. The copyright holder for this preprint (which was not certified by peer review) is the author/funder, who has granted bioRxiv a license to display the preprint in perpetuity. It is made available under aCC-BY 4.0 International license.

Figure S3, Lu et al

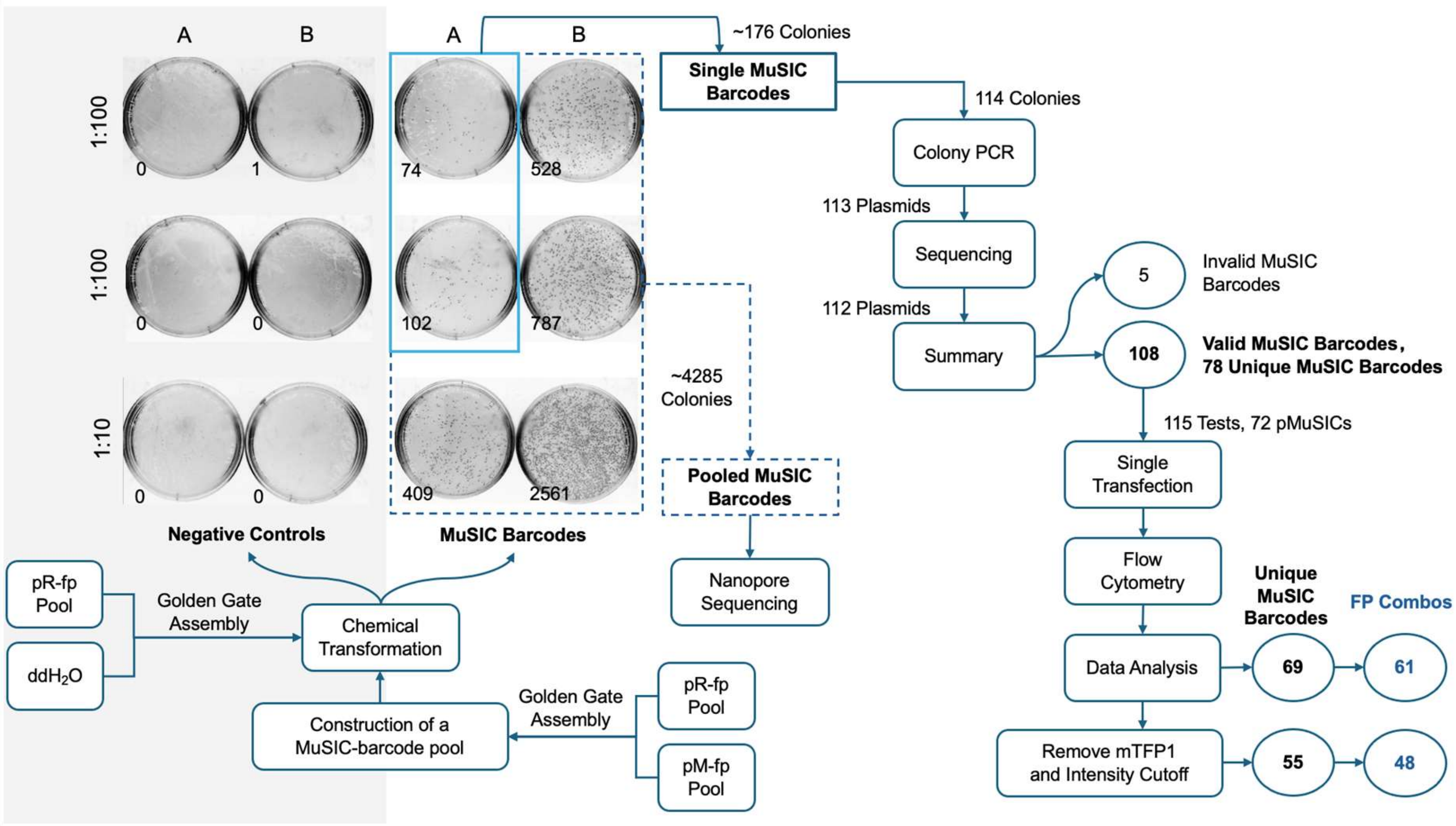

Figure S3. Workflow for Constructing a MuSIC Barcode Pool and Analyzing Single MuSIC Barcodes. This flowchart outlines the process of constructing a MusIC barcode pool through chemical transformation, generating single MuSIC barcodes for flow cytometry analysis and pooled MuSIC barcodes for nanopore sequencing. To better isolate single colonies, we performed duplicate transformations using a 1:100 dilution of the GoldenGate assembly product. To maximize the colonies without compromising the transformation efficiency, a 1:10 dilution was also performed. We used the ddH_2O in place of the insert pool as the negative control. The number in the bottom left corner of each plate represents the colony count. More than 4000 colonies from the MuSIC-barcode plates, highlighted by the navy dotted square, were scraped and pooled to create the MuSIC barcode library for nanopore sequencing. In the skyblue solid square, 114 out of 176 single colonies from the duplicate plates (A) from the 1:100 dilution were selected and screened by colony PCR. Positive candidates were further verified by sequencing (*PlasmidSaurus*). Among 112 positive pMuSICs, 108 were confirmed to contain different probes and thus were considered valid MuSIC barcodes. We chose 69 unique barcodes for flow cytometry. After removing mTFP1 and applying an intensity cutoff at 10^4 to exclude cells with low fluorescence intensity, 55 unique barcodes remained, resulting in a total of 48 distinct fluorescent protein combinations.

bioRxiv preprint doi: https://doi.org/10.1101/2024.10.23.619855; this version posted October 24, 2024. The copyright holder for this preprint (which was not certified by peer review) is the author/funder, who has granted bioRxiv a license to display the preprint in perpetuity. It is made available under aCC-BY 4.0 International license.

Figure S4, Lu et al

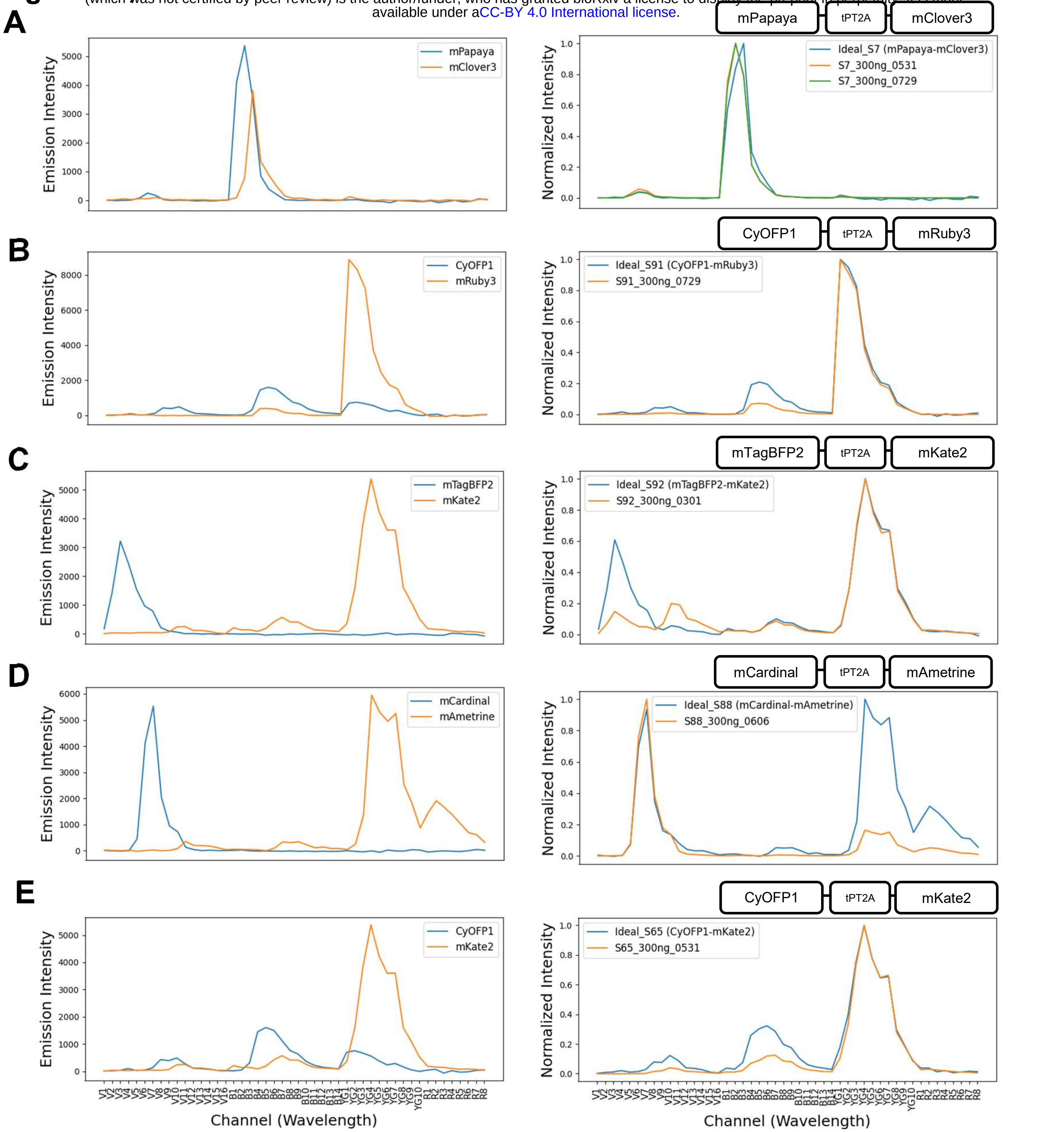

Figure S4. Spectra Related to Difficult Barcodes. (left) Raw intensity median spectra of individual fluorescent proteins in HEK293 cells. The raw intensity helps inform on brightness differences (if applicable). (right) Normalized intensity median spectra of "ideal" and measured median spectra in HEK293 cells. The "ideal" spectra is that expected from linear combination of the two individual fluorescent proteins. The order of each barcode from the plasmid is depicted at its top right. The normalized intensity helps to compare between ideal and measured—brightness differences are built-in. (**A**) mClover3-mPapaya. (**B**) CyOFP1-mRuby. (**C**) mTagBFP2-mKate2. (**D**) mCardinal-mAmetrine. (**E**) CyOFP1-mKate2.

bioRxiv preprint doi: https://doi.org/10.1101/2024.10.23.619855; this version posted October 24, 2024. The copyright holder for this preprint (which was not certified by peer review) is the author/funder, who has granted bioRxiv a license to display the preprint in perpetuity. It is made available under aCC-BY 4.0 International license.

Figure S5, Lu et al

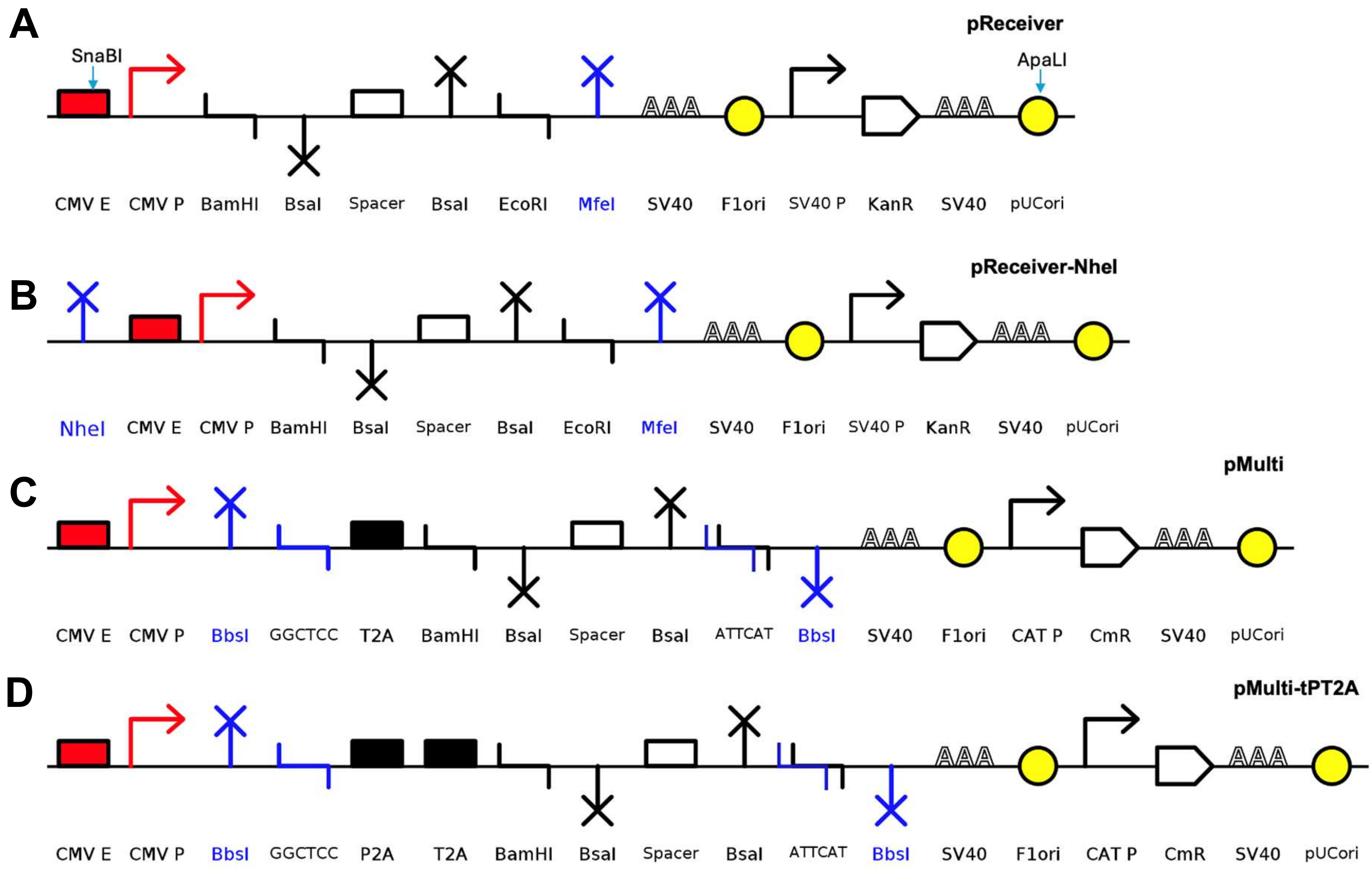

Figure S5. Schematic Representations of Plasmid Backbones. The diagrams illustrate the key features of the plasmid backbones, including the origin of the replicates(ori), antibiotic resistance markers (KanR or CmR), CMV promoter regions （CMV Enhancer/CMV Promoter), and BsaI-Spacer-BsaI cassette for fluorescent protein (fp) insertion. The schematic representations were generated using the SBOLcanvas online tool (https://sbolcanvas.org/canvas/). (**A**) The SnaBI and ApaLI restriction sites (sky blue arrows) of pReceiver were used to generate pReceiver-NheI via overlap extension PCR. (**B**) An NheI was inserted upstream of the CMV promoter region in pReceiver-NheI, along with an MfeI site before the poly A tail, to facilitate the generation of sequencing fragments for subsequent nanopore sequencing. (**C**) A pair of BbsI sites was introduced before the T2A and after the BsaI-Spacer-BsaI fp insertion cassette, allowing the T2A-fp fragment to be inserted into pR-fp probe to create MuSIC barcodes. (**D**) The T2A sequence was replaced by a tandem P2A-T2A to enhance the cleavage efficiency and prevent the potential FRET interference. The paired BsaI restriction sites were oriented in opposite directions, as were the paired BbsI restriction sites in (**C)** and (**D)**.

bioRxiv preprint doi: https://doi.org/10.1101/2024.10.23.619855; this version posted October 24, 2024. The copyright holder for this preprint (which was not certified by peer review) is the author/funder, who has granted bioRxiv a license to display the preprint in perpetuity. It is made available under aCC-BY 4.0 International license.

Figure S6, Lu et al

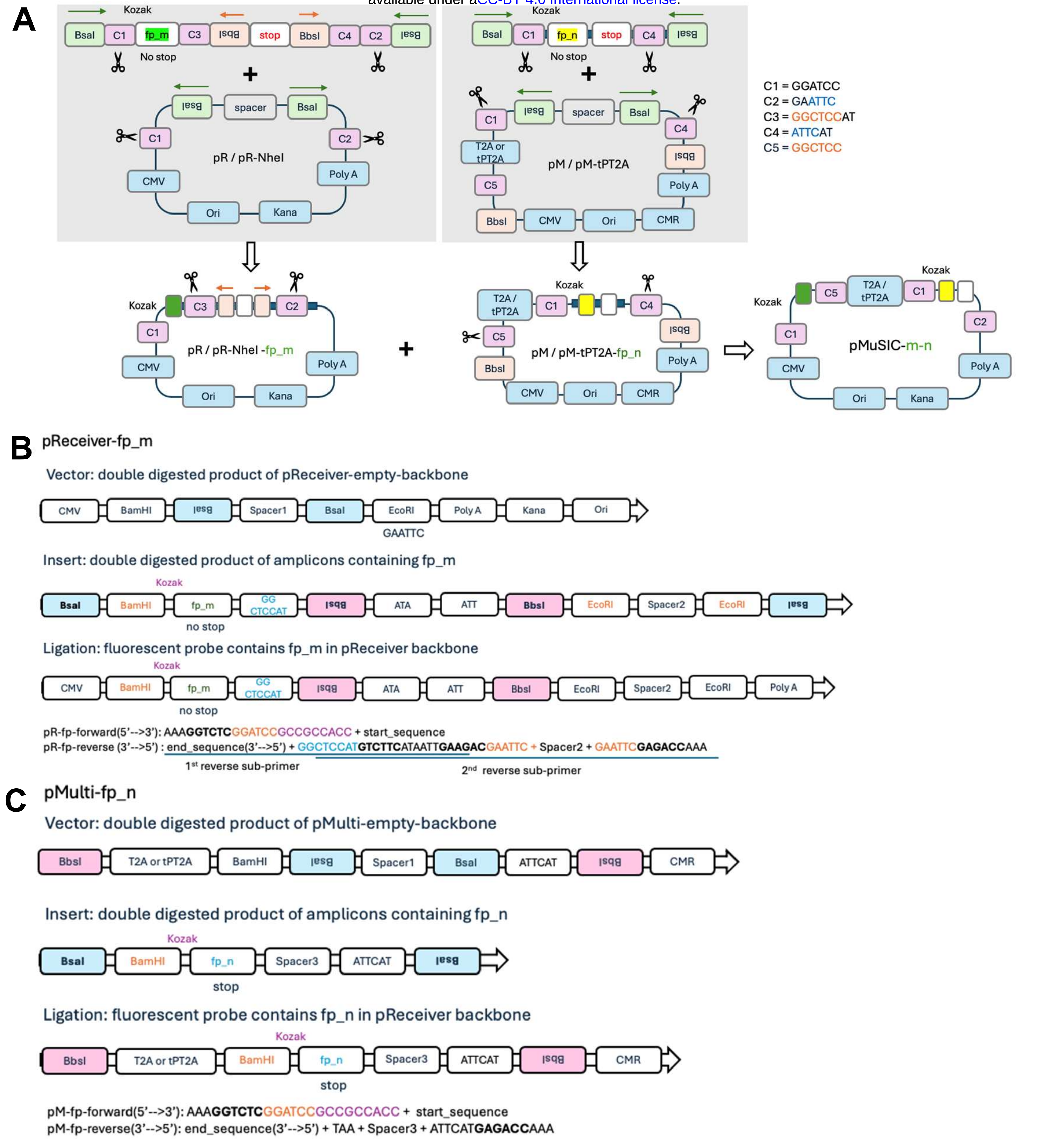

Figure S6. pMuSIC Construction. (**A**) Constructing pMuSIC by Goldengate. First, the BsaI-Spacer-BsaI cassettes in pR were removed by BbsI digestion, and the PCR products containing a fluorescent protein (fp, index *m*) and BbsI-TAA-BbsI cassette (stop), were inserted to create the vector plasmid pR-NheI-fp_m-BbsI-TAA-BbsI. Similarly, pM underwent BsaI digestion for the fp insertion (fp, index *n*), resulting in the insert plasmids pM-fp_n. Finally, both vector and insert plasmids were digested at BbsI sites to generate pMuSICs containing the MuSIC barcode (fp_m-2A-fp_n). (**B-C**) Primer designs to amplify the fp inserts for both the vector and insert plasmids, respectively. The sequences of all 18 fps were used as templates (**Table S5**), with their start and end sequences listed in **Table S6**, along with the sequences of the spacers.

bioRxiv preprint doi: https://doi.org/10.1101/2024.10.23.619855; this version posted October 24, 2024. The copyright holder for this preprint (which was not certified by peer review) is the author/funder, who has granted bioRxiv a license to display the preprint in perpetuity. It is made available under aCC-BY 4.0 International license.

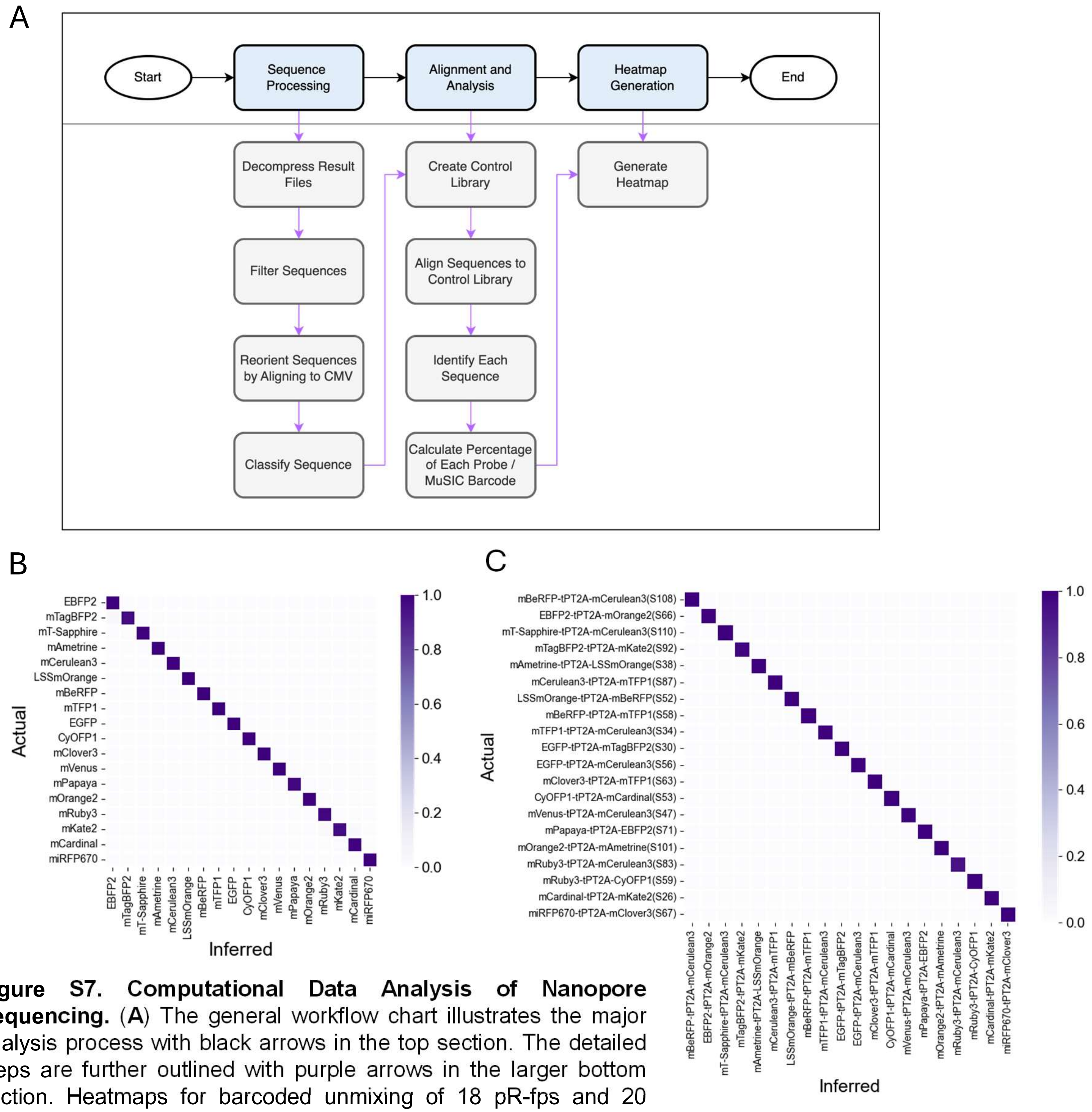

Figure S7. Computational Data Analysis of Nanopore Sequencing. (**A**) The general workflow chart illustrates the major analysis process with black arrows in the top section. The detailed steps are further outlined with purple arrows in the larger bottom section. Heatmaps for barcoded unmixing of 18 pR-fps and 20 sequenced pMuSICs were generated as shown in (**B**) and (**C**). Color bar denotes TPR.

bioRxiv preprint doi: https://doi.org/10.1101/2024.10.23.619855; this version posted October 24, 2024. The copyright holder for this preprint (which was not certified by peer review) is the author/funder, who has granted bioRxiv a license to display the preprint in perpetuity. It is made available under aCC-BY 4.0 International license.

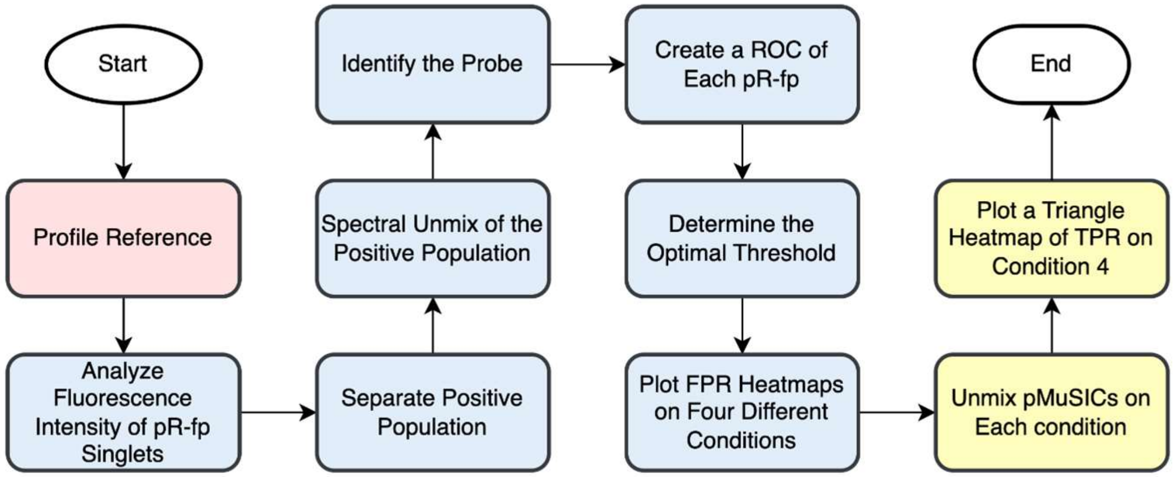

Figure S8. Workflow Chart of Spectral Unmixing Data Analysis. This chart demonstrates the step-by-step process of spectral unmixing data analysis, from reference profiling of the manually gated positive population of singly transfected pR-fp cells (pink box), analyzing of the entire population of pR-fp singlets (blue boxes), to the unmixing of the positive population of singly transfected pMuSIC cells (yellow boxes).
